# A novel combination therapy targeting ubiquitin-specific protease 5 in MYCN-driven neuroblastoma

**DOI:** 10.1038/s41388-021-01712-w

**Published:** 2021-03-03

**Authors:** Belamy B. Cheung, Ane Kleynhans, Rituparna Mittra, Patrick Y. Kim, Jessica K. Holien, Zsuzsanna Nagy, Olivia C. Ciampa, Janith A. Seneviratne, Chelsea Mayoh, Mukesh Raipuria, Satyanarayana Gadde, Hassina Massudi, Iris Poh Ling Wong, Owen Tan, Andrew Gong, Aldwin Suryano, Sonya M. Diakiw, Bing Liu, Greg M. Arndt, Tao Liu, Naresh Kumar, Olle Sangfelt, Shizhen Zhu, Murray D. Norris, Michelle Haber, Daniel R. Carter, Michael W. Parker, Glenn M. Marshall

**Affiliations:** 1 Children’s Cancer Institute, Lowy Cancer Research Centre, UNSW Sydney, Sydney, NSW Australia; 2 School of Women’s and Children’s Health, UNSW Sydney, Sydney, NSW Australia; 3 ACRF Rational Drug Discovery Centre, St. Vincent’s Institute of Medical Research, Fitzroy, VIC Australia; 4 School of Science, College of Science, Engineering and Health, RMIT University, Melbourne, VIC Australia; 5 School of Chemistry, UNSW Sydney, Sydney, NSW Australia; 6 Department of Cell and Molecular Biology, Karolinska Institute, Stockholm, Sweden; 7 Department of Biochemistry and Molecular Biology, Cancer Center and Center for Individualized Medicine, Mayo Clinic, Rochester, MN USA; 8 University of New South Wales Centre for Childhood Cancer Research, Sydney, NSW Australia; 9 School of Biomedical Engineering, University of Technology Sydney, Sydney, NSW Australia; 10 Bio21 Molecular Science and Biotechnology Institute, The University of Melbourne, Parkville, VIC Australia; 11 Kids Cancer Centre, Sydney Children’s Hospital, Randwick, NSW Australia

**Keywords:** Paediatric cancer, High-throughput screening, Target identification

## Abstract

Histone deacetylase (HDAC) inhibitors are effective in MYCN-driven cancers, because of a unique need for HDAC recruitment by the MYCN oncogenic signal. However, HDAC inhibitors are much more effective in combination with other anti-cancer agents. To identify novel compounds which act synergistically with HDAC inhibitor, such as suberanoyl hydroxamic acid (SAHA), we performed a cell-based, high-throughput drug screen of 10,560 small molecule compounds from a drug-like diversity library and identified a small molecule compound (SE486-11) which synergistically enhanced the cytotoxic effects of SAHA. Effects of drug combinations on cell viability, proliferation, apoptosis and colony forming were assessed in a panel of neuroblastoma cell lines. Treatment with SAHA and SE486-11 increased MYCN ubiquitination and degradation, and markedly inhibited tumorigenesis in neuroblastoma xenografts, and, *MYCN* transgenic zebrafish and mice. The combination reduced ubiquitin-specific protease 5 (USP5) levels and increased unanchored polyubiquitin chains. Overexpression of USP5 rescued neuroblastoma cells from the cytopathic effects of the combination and reduced unanchored polyubiquitin, suggesting USP5 is a therapeutic target of the combination. SAHA and SE486-11 directly bound to USP5 and the drug combination exhibited a 100-fold higher binding to USP5 than individual drugs alone in microscale thermophoresis assays. MYCN bound to the USP5 promoter and induced USP5 gene expression suggesting that USP5 and MYCN expression created a forward positive feedback loop in neuroblastoma cells. Thus, USP5 acts as an oncogenic cofactor with MYCN in neuroblastoma and the novel combination of HDAC inhibitor with SE486-11 represents a novel therapeutic approach for the treatment of MYCN-driven neuroblastoma.

## Introduction

The oncoproteins, MYCN, MYC, and MYCL are short-lived transcription factors that are overexpressed or deregulated in more than half of all human cancer, making them attractive therapeutic targets [1, 2]. The design of MYC inhibitors has been hampered by the lack of globular functional MYC domains or deep protein ‘pockets’ for drug design [3]. Moreover, MYC inhibitors carry a heightened potential for side-effects due to the dependency of most normal cells on transient MYC expression at entry into the cell cycle [4]. This normal cell dependency on rapid increases in MYC levels is exploited by cancer cells through perturbations of the normal cellular mechanisms which destabilise MYC proteins. *MYCN* is amplified or overexpressed in one third of children with neuroblastoma, and, is a driver in several other human cancer types [1, 5, 6]. We and others have shown that increased MYCN protein stability is acquired by neuroblastoma cells, even in the presence of *MYCN* amplification, by multiple feed-forward expression loops involving MYCN trans-activation and -repression target genes [6–8]. MYCN protein stability is tightly regulated in normal cells through several post-translational modifications, MYCN binding proteins, and the ubiquitin ligase SCF-FBXW7 [9].

Histone deacetylases (HDACs) are key regulators of gene expression in normal and malignant cells which repress transcription through the post-translational histone modification of lysine deacetylation [10]. HDAC inhibitor drugs induce a broad range of anti-cancer effects including cell cycle arrest, differentiation, apoptosis, and anti-angiogenic effects with low toxicity to normal cells [11]. HDAC inhibitors have demonstrated strong efficacy in pre-clinical models of MYC and MYCN-driven cancer [12, 13]. Despite the effectiveness of HDAC inhibitors in pre-clinical models only four HDAC inhibitors, including suberanoyl hydroxamic acid (SAHA) are currently approved by the US Food and Drug Administration for the treatment of T-cell lymphoma and multiple myeloma [14]. HDAC inhibitors have significant clinical limitations, including low concentrations in solid tumours, cardiac toxicity, and limited efficacy as single agents, which is hindering their progress toward the clinic [15, 16].

Here, we identified a novel small molecule, SE486-11, from a random drug library screen which enhanced the cytopathic effects of SAHA across a panel of neuroblastoma cell lines and completely blocked tumour growth in *MYCN* transgenic mice and zebrafish. The SAHA + SE486-11 combination repressed MYCN protein levels indirectly through effects on the ubiquitin-specific protease (USP), USP5. Our results suggest USP5 protects MYCN protein from ubiquitin-mediated degradation by lowering unanchored polyubiquitin chain levels in neuroblastoma cells, an effect which can be reversed by direct binding of both drugs to USP5. Together these data highlight an unexpected vulnerability of MYCN protein to unanchored polyubiquitin chain levels in neuroblastoma cells and suggest a novel therapeutic strategy.

## Results

### SE486-11 synergistically enhances SAHA cytotoxicity for neuroblastoma cells

**Fig. 1:**
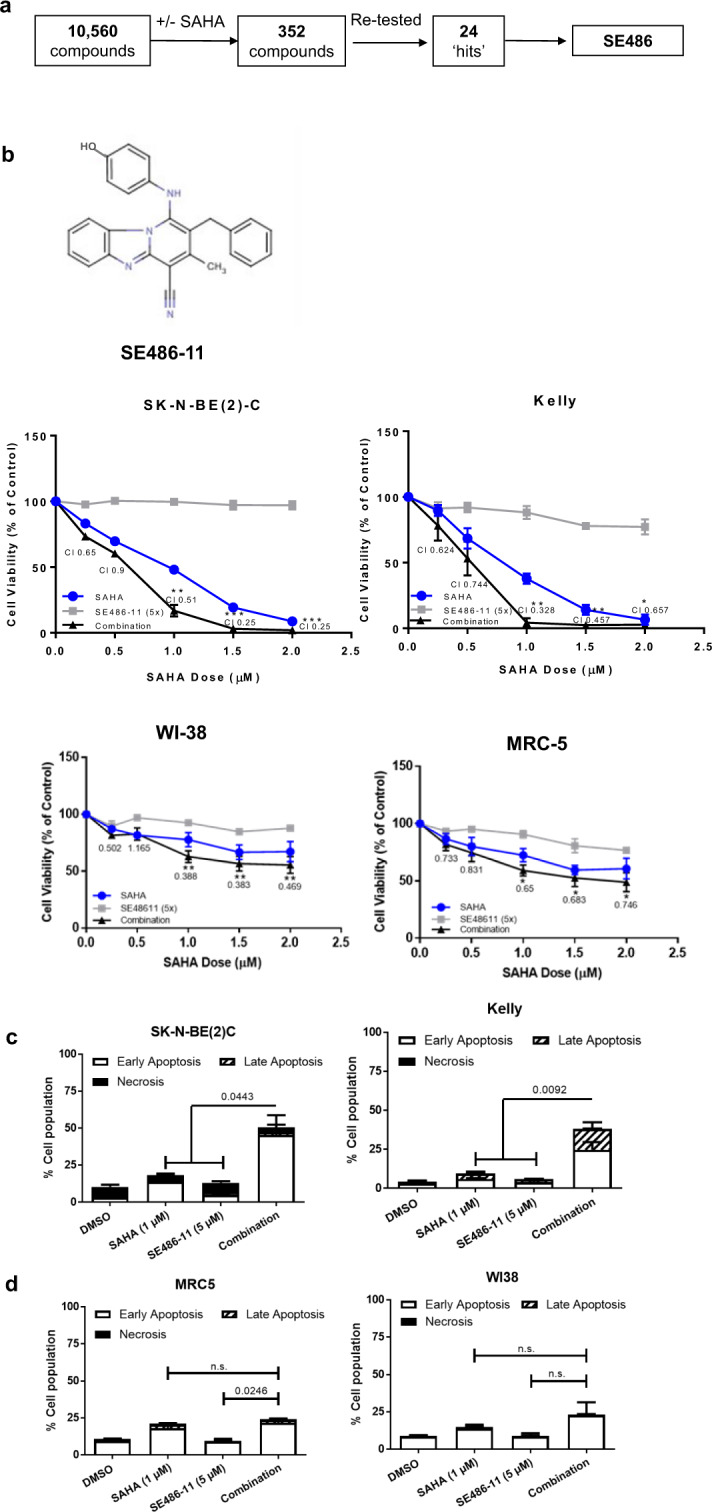

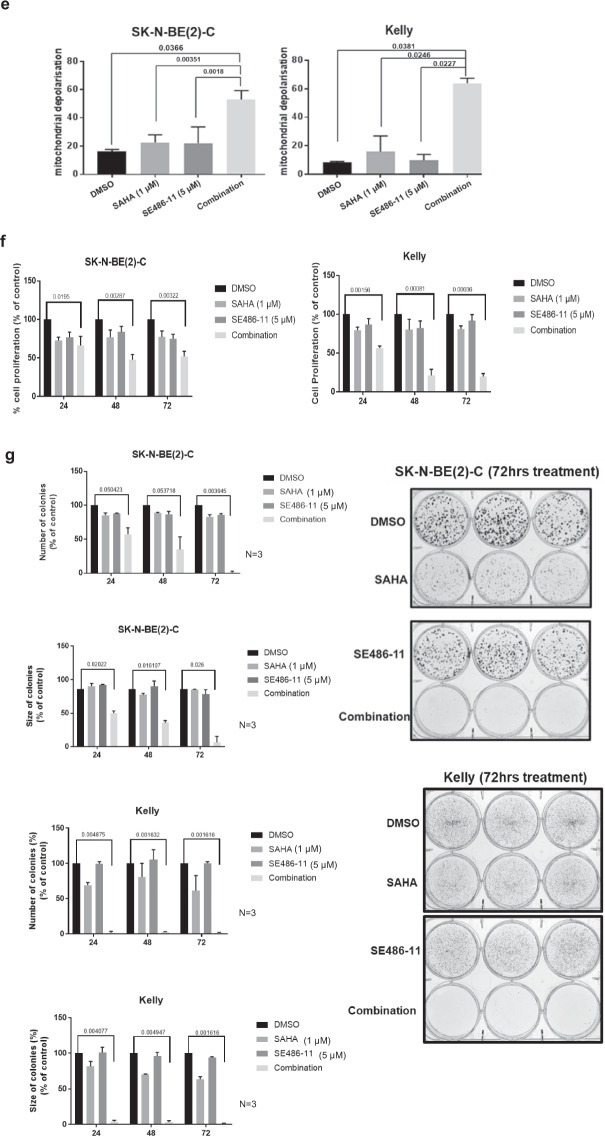
Combinatorial drug screening identified a novel compound that synergistically enhanced the cytotoxicity of suberanoyl hydroxamic acid (SAHA). **a** The steps in screening process of 10,560 compounds from the Walter and Eliza Hall Institute (WEHI) diverse compound library on the SAHA-resistant MDA-MB-231 breast cancer cell line. This identified 24 hits that showed less than 40% viability when used in combination with SAHA, but greater than 70% cell viability when used alone. **b** Top panel: The chemical structure of the identified hit compound, SE486-11. Lower panel: The cell viability of neuroblastoma SK-N-BE(2)-C and Kelly cells, and human fibroblasts WI-38 and MRC-5 cells were measured by Alamar Blue assays after treatment with DMSO, SAHA, SE486-11, or the combination, at different concentrations (SAHA: SE486-11 ratio: 1:5) for 72 h. The cell viability was calculated and displayed as the percentage of DMSO control for single agents and the combination. The resulting combination index (CI) theorem of Chou–Talalay showed quantitative definition for additive effect (CI = 1), synergism (CI < 1), and antagonism (CI > 1) in drug combinations [42]. Significance was determined from three independent experiments. **c** Apoptotic and necrotic effects of the single agents and combination treatment was determined following pre-treatment of SK-N-BE(2)-C and Kelly neuroblastoma cells for 72 h before collecting the cells and staining with Annexin V/7-AAD and loss of staining was detected by FACS analysis. A summary of results is shown as the percentage of cells in early or late apoptosis, and necrosis as histograms. Significance was determined from three independent experiments. **d** Apoptotic and necrotic effects of the single agents and combination treatment was determined following pre-treatment of MRC-5 and WI-38 cells for 72 h before collecting the cells and staining with Annexin V/7-AAD and loss of staining was detected by FACS analysis. **e** The mitochondrial polarisation of SK-N-BE(2)-C and Kelly cells after 48 h of treatment with DMSO, single agents or combination therapy was determined using the JC-1 assay. Cells were collected and stained with JC-1 dye and the mitochondrial membrane potential determined following FACS analysis. The bar graph indicates the averaged data, mean ± SD, as the percentage of cells with lowered red fluorescence, measuring mitochondrial depolarisation. Significance (**p < 0.01) was determined from at least three independent experiments. **f** Effect of DMSO, single agents, and combination therapy (1 µM SAHA + 5 µM SE486-11) on cellular proliferation of SK-N-BE(2)-C and Kelly neuroblastoma cell lines after 24, 48, and 72 h of treatment was determined by measuring BrdU incorporation. Data are presented as mean ± SD percentage of cell proliferation after normalisation to the control untreated cells. Significance (*p < 0.001, **p < 0.0001) was determined from at least three independent experiments. **g** In vitro clonogenic growth assay performed in SK-N-BE(2)-C and Kelly cells by counting the number of colonies and the sizes of colonies after 21 days post cell plating and treatment with DMSO, single agents and combination therapy (1 µM SAHA + 5 µM SE486-11). Histogram represents mean ± SD of the number of colonies after 24, 48, and 72 h of treatment and growing for 21 days. The significance (*p < 0.01, **p < 0.0001; ***p < 0.00001) was determined after three independent experiments. Representative examples of stained colonies are shown for each treatment in both cell lines.

Pre-clinical studies have shown that the HDAC inhibitor, SAHA, inhibits the growth of neuroblastoma cells in vivo [17]. A phase I/II clinical trial of SAHA as a single agent in children with solid tumours (neuroblastoma) showed no responses to SAHA when given as a single agent. However, when SAHA in combination with 13-*cis*-retinoic acid was given to patients, a response was observed [18]. We have performed a search on Genomics of Drug Sensitivity in Cancer database, and showed that invasive breast carcinoma cell lines are more SAHA-resistant compared to other cancer cell lines (Fig. S1a). Therefore, to identify novel compounds which act synergistically with SAHA, we performed a cell-based, high-throughput drug screen using a MYC-expressing breast cancer cell line (MDA-MB-231) [19] with relatively high resistance to SAHA (IC50 4.6 µM) compared to neuroblastoma cell lines. We treated these cells in an in vitro screen of 10,560 small molecules from a drug-like diversity library, at a dose of a 10 µM combined with 1.9 µM (IC20 for MDA-MB-231 cells) SAHA (Fig. 1a). A total of 352 compounds were selected and screened in the presence or absence of 1.9 µM SAHA. In the presence of 1.9 μM SAHA, 88% of the 352 compounds led to MDA-MB-231 cell viability of <40%, showing that the large-scale screen conditions were robust. Twenty-four of the 352 compounds caused: (i) cell viability of <40% in the presence of SAHA; (ii) cell viability of >70% in the absence of SAHA; and, (iii) at least a 55% difference between cell viabilities measured in the presence and absence of SAHA. These stringency criteria produced a hit rate of 0.23% (24/10,560 compounds) (Table S1 and Fig. 1a). A 48 h cytotoxicity screen of 12 structural analogues with more than 80% structural similarity to the most effective compound from the primary screen, SE486, in two human neuroblastoma cell lines (SK-N-BE(2)C and Kelly) identified a pyridobenzimidazole analogue, SE486-11 (2-benzyl-1-[(4-hydroxyphenyl)amino]-3-methylpyrido[1,2-a]benzimidazole-4-carbonitrile) as the most potent agent (Fig. S1b). A secondary screen was carried out to test the efficacy of the combination across various cancer cell lines (Fig. S1c). This SAHA + SE486-11 combination therapy screen revealed that MYCN-amplified neuroblastoma cell lines were the most sensitive cell lines. We, therefore, proceeded to focus our study on MYCN-amplified neuroblastoma cell lines. The combination of SE486-11 and SAHA was highly synergistic against neuroblastoma MYCN-amplified SK-N-BE(2)-C and Kelly cell lines after 72 h treatment, but had limited cytotoxicity against normal human fibroblasts, WI-38 and MRC-5 cells (Fig. 1b). To determine if the synergy of the combination therapy is SAHA-specific or shared by other hydroxamate-based HDAC inhibitors, we treated SK-N-BE(2)-C and Kelly cells with SE486-11 in combination with Panobinostat. The combination of SE486-11 and Panobinostat was also highly synergistic at a range of concentrations against SK-N-BE(2)-C and Kelly cell lines after 72 h of treatment (Fig. S1d).

The combination of SAHA + SE486-11 synergistically induced apoptosis in neuroblastoma cells after 72 h (Fig. 1c; Fig. S1e, f), but not MRC-5 and WI-38 normal fibroblasts (Fig. 1d; Fig. S1g, h), as measured by flow cytometry following Annexin V-7AAD staining. Only the combination therapy activated apoptosis by cleaving caspase 3, while a pan-caspase inhibitor blocked the effects of combination therapy on cell viability (Fig. S1i, j). The combination treatment for 48 h increased mitochondrial depolarisation by JC-1 staining in *MYCN*-amplified neuroblastoma SK-N-BE(2)-C and Kelly cells (Fig. 1e; Fig. S1k, l). To determine whether the SAHA + SE486-11 combination could inhibit cell proliferation in vitro, we performed BrdU incorporation and colony forming assays. The combination significantly reduced cell proliferation as measured by BrdU incorporation (Fig. 1f) and reduced colony number and size in SK-N-BE(2)-C and Kelly cells (Fig. 1g). These data suggest that the markedly reduced cell viability resulting from combination treatment is due to both reduced proliferation and an increase in apoptosis.

### The combination of SAHA + SE486-11 inhibits tumour growth of human neuroblastoma cell line xenografts, *MYCN* transgenic mice, and zebrafish

**Fig. 2:**
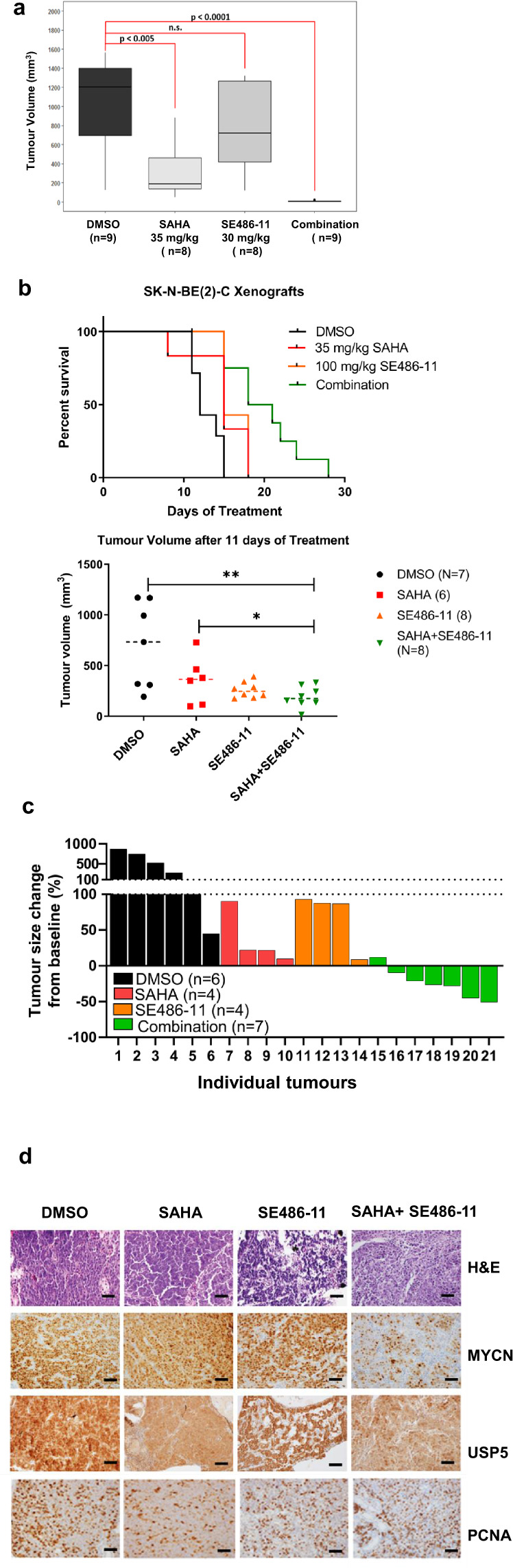
In vivo tumour formation in neuroblastoma-bearing transgenic *TH-MYCN*^*+/+*^ mice and MYCN;GFP zebrafish. **a** *TH-MYCN*^*+/+*^ mice were treated with the single agents or combination therapy from age 3 weeks old when tumours are first palpable, for a period of 21 days using a 5 days on and 2 days off schedule. Tumour volume (mm^3^) was measured at the end of the treatment period. **b** SK-N-BE(2)-C cells were injected subcutaneously into the flank of BALB/c nude mice (*n* = 7–9 mice/group). When the tumour size had reached 4 mm, mice were treated with DMSO (control), SAHA alone, SE486-11 alone, and SAHA + SE486-11 for 5 consecutive days until the tumour reached 1000 mm^3^. Percentage survival was calculated using the Log-rank (Mantel–Cox) test. Tumour volume was measured at day 11 of treatment of human neuroblastoma cell xenografts with SAHA, SE486-11, or SAHA + SE486-11. *P* values were obtained using unpaired *t*-test, two tail. *P < 0.05, **P < 0.01. **c** A waterfall plot displaying the % difference in tumour size from Day 1 of treatment to Day 7 for every single tumour treated with DMSO, SAHA, SE486-11, or combination in neuroblastoma-bearing MYCN-transgenic zebrafish. The size of the EGFP-positive tumour was measured using ImageJ software. **d** Histopathologic and immunohistochemical analyses of MYCN;GFP zebrafish tumours treated with DMSO, SAHA, SE486-11, or combination drug treatment. Top to bottom: Neuroblastoma tumour sections (×40 magnification) immunohistochemically stained for Haematoxylin & Eosin (H&E), MYCN, USP5, and proliferating cell nuclear antigen (PCNA).

Next, we tested the effect of combination therapy in neuroblastoma-bearing transgenic *TH-MYCN* mice. Homozygote *TH-MYCN*^+/+^ transgenic mice develop neuroblastoma in paravertebral and coeliac ganglia with most of the features of the human disease at postnatal 6–7 weeks in 100% of mice [20]. *TH-MYCN*^+/+^ mice were treated with intraperitoneal injections of either DMSO alone, 35 mg/kg of SAHA, 30 mg/kg of SE486-11, or a combination of SAHA + SE486-11 at the single agent doses from 3 weeks of age. Treatments were started when abdominal tumours are first palpable at 3 weeks of age, then extended for 3 weeks, using 5 days on and 2 days off treatment schedule. The combination therapy markedly reduced tumour formation compared with partial inhibition of tumorigenicity with either agent alone. The combination treatment was highly potent, in that no tumour tissue remained for further histopathological analysis. The body weight of the mice did not change during the treatment, suggesting that there were no serious treatment side effects (Fig. 2a; Fig. S2a, b). The antitumor activity of SAHA, SE486-11, and SAHA + SE486-11, against neuroblastoma cell line xenografts was assessed by effects on tumour volume and overall survival (Fig. 2b). Survival of SK-N-BE(2)-C xenograft mice were calculated at endpoint, when tumour size reached 1000 mm^3^. Overall survival was greater in the SAHA + SE486-11 group compared with controls (DMSO) (*p* < 0.0006), SAHA alone (*p* < 0.056), and SE486-11 alone (*p* < 0.003). Tumour volume after 11 days of treatment were significantly lower in SAHA + SE486-11 group compared with controls (DMSO) (*p* < 0.005) and with SAHA alone (*p* < 0.044) (Fig. 2b; Fig. S2c, d).

In addition, neuroblastoma-bearing *MYCN* transgenic zebrafish at 0.5–1.5 years of age overexpressing dβh:EGFP-MYCN [21], were treated with 2 μl of vehicle (100% DMSO), 1 μl of SAHA (21 mg/kg), 1 μl of SE486-11 (30 mg/kg), or 1 μl of SAHA plus 1 μl of SE486-11 by oral gavage [22]. The GFP-positive tumours naturally varied in size among the cohort of fish tested. However, when comparing day 1 treated fish with day 7 treated fish within each treatment group, only the combination therapy showed a significant reduction in the GFP-positive tumour size, compared to DMSO and the single agents (Fig. 2c; Fig. S2e). Combination drug treatment with SAHA and SE486-11 reduced MYCN, USP5 and PCNA (proliferation marker) protein levels in neuroblastoma-bearing *MYCN*;GFP zebrafish compared to DMSO treated controls (Fig. 2d; Fig. S2f).

### SAHA + SE486-11 therapy reduces MYCN protein stability through increased MYCN ubiquitination and proteasomal degradation

**Fig. 3:**
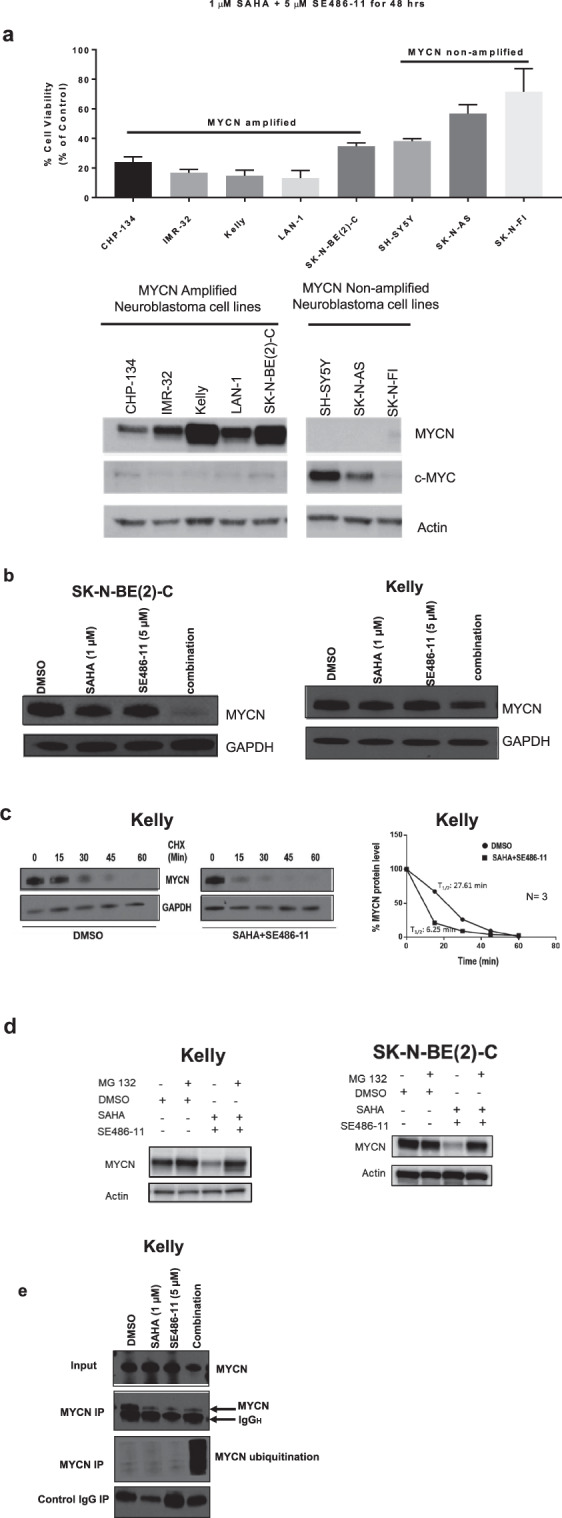
Combination therapy significantly reduced MYCN protein expression and stability, and, increased ubiquitination. **a** Top panel: cell viability was measured using the Alamar Blue assay across a panel of MYCN non-amplified and MYCN-amplified cell lines after 48 h of combination therapy (1 µM SAHA + 5 µM SE486-11). Results are represented as the mean ± SD for at least three independent experiments. Bottom panel: Immunoblot analysis with MYCN, c-Myc, and actin antibodies against whole cell protein lysates from both MYCN non-amplified and MYCN-amplified cell lines without combination treatment. **b** Kelly and SK-N-BE(2)-C neuroblastoma cell lines were treated with either DMSO, single agents, or combination therapy (1 µM SAHA + 5 µM SE486-11) for 24 h. Cell protein lysates were collected, and representative immunoblots of MYCN expression using a MYCN antibody. **c** Representative immunoblots from cycloheximide (CHX) chase assays after 24 h of DMSO or combination treatment (1 µM SAHA + 5 µM SE486-11) of Kelly cells. Cells were then treated with 100 µg/ml CHX for up to 60 min followed by immunoblotting and densitometric analysis to determine the protein half-life of MYCN relative to no CHX. **d** Immunoblot analysis with a MYCN antibody against whole cell protein lysates from Kelly and SK-N-BE(2)-C cells treated with combination therapy for 24 h, and then treated with 30 µM of the proteasome inhibitor, MG-132, for 4 h. **e** Representative immunoblot of Kelly cells treated with DMSO, single agents, or combination therapy (1 µM SAHA + 5 µM SE486-11) for 48 h, before cell protein lysates were collected and endogenous MYCN was immunoprecipitated using a MYCN antibody. Ubiquitination was detected in each pull-down using a Ubiquitin antibody.

To understand the mechanism of action of the SAHA + SE486-11 combination, we examined the cytotoxicity of combination therapy (1 µM SAHA + 5 µM SE486-11) for a panel of human neuroblastoma cell lines after 48 h of continuous drug exposure, subdivided for the presence or absence of *MYCN* amplification. We performed immunoblot analysis to confirm MYCN and c-MYC protein expression. We found that the *MYCN*-amplified neuroblastoma cell lines (CHP134, IMR-32, Kelly, LAN-1 and SK-N-BE(2)-C) with high MYCN expression had the greatest sensitivity to the cytotoxic effects of the combination treatment in comparison with *MYCN* non-amplified cell lines (SH-SY5Y, SK-N-AS, and SK-N-FI) (Fig. 3a). These data suggested that expression of the MYCN oncoprotein may be a target of the combination therapy. We next showed that SAHA + SE486-11 markedly reduced MYCN protein expression after 24 h of treatment in *MYCN*-amplified cells in vitro (Fig. 3b), whereas there was no effect on MYC protein levels in *MYCN* non-amplified SH-SY5Y and SK-N-AS cells (Fig. S3a). Since combination therapy had no significant effect on *MYCN* mRNA levels assessed by qPCR comparing treated and untreated in neuroblastoma cells (Fig. S3b), we performed a cycloheximide chase experiment to assess the effect of combination therapy on MYCN protein half-life in *MYCN*-amplified Kelly cells (Fig. 3c). We demonstrated a dramatic 4-fold reduction in MYCN protein half-life following combination therapy.

To evaluate whether combination therapy increased MYCN degradation through ubiquitination and the proteasomal pathway, we treated neuroblastoma cells with combination therapy for 24 h and then added the proteasome inhibitor MG-132 (30 µM) for 4 h. We found that MG132 increased MYCN protein levels in the presence of combination therapy (Fig. 3d). When immunoprecipitated endogenous MYCN from *MYCN*-amplified neuroblastoma cells was immunoblotted with a ubiquitin antibody, we found that combination therapy markedly increased MYCN ubiquitination (Fig. 3e). These data indicated that the cytopathic effects of combination therapy on neuroblastoma cells were mediated in part by enhanced MYCN ubiquitination and protein degradation.

### USP5 is a therapeutic target of combination therapy

To better understand the mechanism by which the combination therapy affected MYCN protein ubiquitination, we performed a comparative Stable Isotope Labelling with Amino acids in Cell culture (SILAC) assay on total cellular protein from neuroblastoma cells treated with either DMSO, SAHA, SE486-11, or the combination. The peak intensities of the heavy and light peptides were compared to determine the changes in protein expression of the identified proteins with combination treatment. The SILAC assay identified 2248 proteins that were downregulated by the combination treatment compared to DMSO. Statistical comparisons were performed for downregulated proteins in combination-treated cells ranked by *P* values (Fig. S4a). Of the top 10 proteins which were significantly downregulated in the presence of SAHA + SE486-11, USP5 (also called UPB5), was the only deubiquitinase identified (*P* value = 0.0071) (Fig. S4a).

**Fig. 4:**
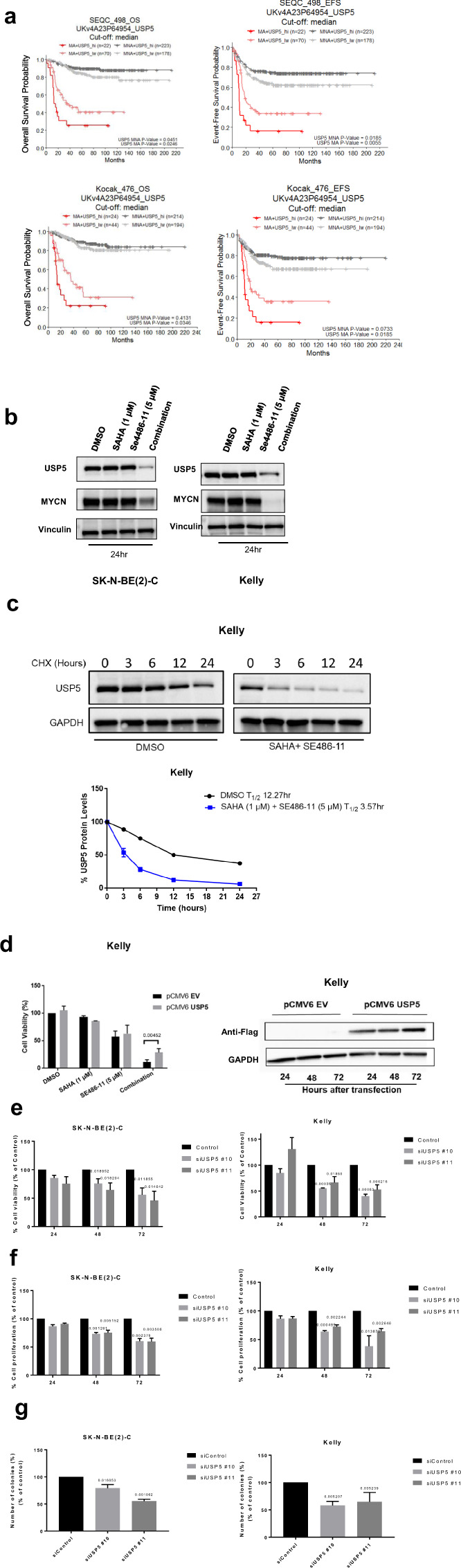
USP5 is a target of combination therapy in neuroblastoma cells. **a** Kaplan–Meier analyses for overall survival (OS) and event-free survival (EFS) of neuroblastoma patients with MYCN amplification (MA) and MYCN non-amplification (MNA), subdivided around the median USP5 mRNA expression level in tumour samples using the publically available SEQC (*n* = 498) and Kocak (*n* = 476) patient datasets. **b** Kelly and SK-N-BE(2)-C neuroblastoma cell lines were treated with either DMSO, single agents, or combination therapy for 48 h. Cell protein lysates were collected, and representative immunoblots of USP5 expression detected using a USP5 or MYCN antibody and a control Vinculin antibody. **c** Representative immunoblots from cycloheximide (CHX) chase assays after DMSO or combination treatment (1 µM SAHA + 5 µM SE486-11) of Kelly cells for 24 h. Cells were then treated with 100 µg/ml CHX for up to 24 h followed by immunoblotting and densitometric analysis to determine the protein half-life of USP5 relative to no CHX. **d** Left panel, overexpression of USP5 in Kelly cells following transient transfection of empty vector or USP5 expression vector for 24 h, followed by 48-h treatment with DMSO, single agents, or combination therapy and measurement of cell viability using the Alamar Blue assay. Significance (**p < 0.001) was determined from at least three independent experiments. Right panel, a representative immunoblot confirming overexpression of USP5 detected by a Flag antibody. **e**–**g** SK-N-BE(2)-C and Kelly neuroblastoma cell lines were transfected with two USP5 siRNAs (siRNA#10 and #11) for 24, 48, and 72 h and the cell viability was measured over time using the Alamar Blue assay (**e**). Cell growth was measured using BrdU incorporation (**f**), and clonogenic colony formation (**g**) after 14 days of treatment. Data are represented as the mean ± SD for three independent experiments and was compared to the control siRNA.

We next examined whether USP5 expression levels had prognostic significance in primary tumour tissue from neuroblastoma patients. High mRNA expression of USP5 had a strong association with poor neuroblastoma patient prognosis in publicly available R2 gene expression datasets (*n* = 469 for Kocak dataset and *n* = 498 for SEQC dataset) for the MYCN-amplified patient subsets (Fig. 4a) [23]. Interestingly, MYCN non-amplified patients with high USP5 expression associated with good prognosis in the SEQC dataset (MNA *p* value = 0.0451 for overall survival and *p* value = 0.0185 for event-free survival). However, there was no statistically significant difference for subgroups with high or low USP5 expression in the Kocak dataset for MYCN non-amplified patients (Fig. 4a). Immunoblotting showed USP5 protein levels were also downregulated after 48 h of combination treatment in MYCN-amplified Kelly and SK-N-BE(2)-C cells, as well as in the MDA-MB-231 breast cancer line (Fig. 4b; Fig. S4b, c). However, there was no reduction in USP5 mRNA levels when Kelly and SK-N-BE(2)-C cells were treat with the combination therapy (Fig. S4d). We next performed a cycloheximide chase assay to investigate whether the combination affected USP5 protein stability. We found a 3.4-fold decrease in USP5 protein half-life when the cells were treated with the combination (Fig. 4c). To determine whether the combination was targeting USP5, we transiently transfected Kelly cells with an empty vector or a USP5 expression vector for 72 h. Twenty-four hours after plating, Kelly cells were transfected, followed by the drug treatment with either single agents or the combination for 48 h. We found that USP5 overexpression partially rescued Kelly cells from the cytopathic effects of combination therapy (Fig. 4d). To determine the effects of USP5 suppression on neuroblastoma cells, we transiently transfected neuroblastoma cell lines with two USP5-specific siRNAs. The siRNA knock-down of USP5 caused a significant decrease in cell viability, cell proliferation, and colony formation in vitro for SK-N-BE(2)-C and Kelly cells (Fig. 4e–g; Fig. S4e, f).

### MYCN induces USP5 gene expression altering the balance of unanchored poly- and mono-ubiquitins

**Fig. 5:**
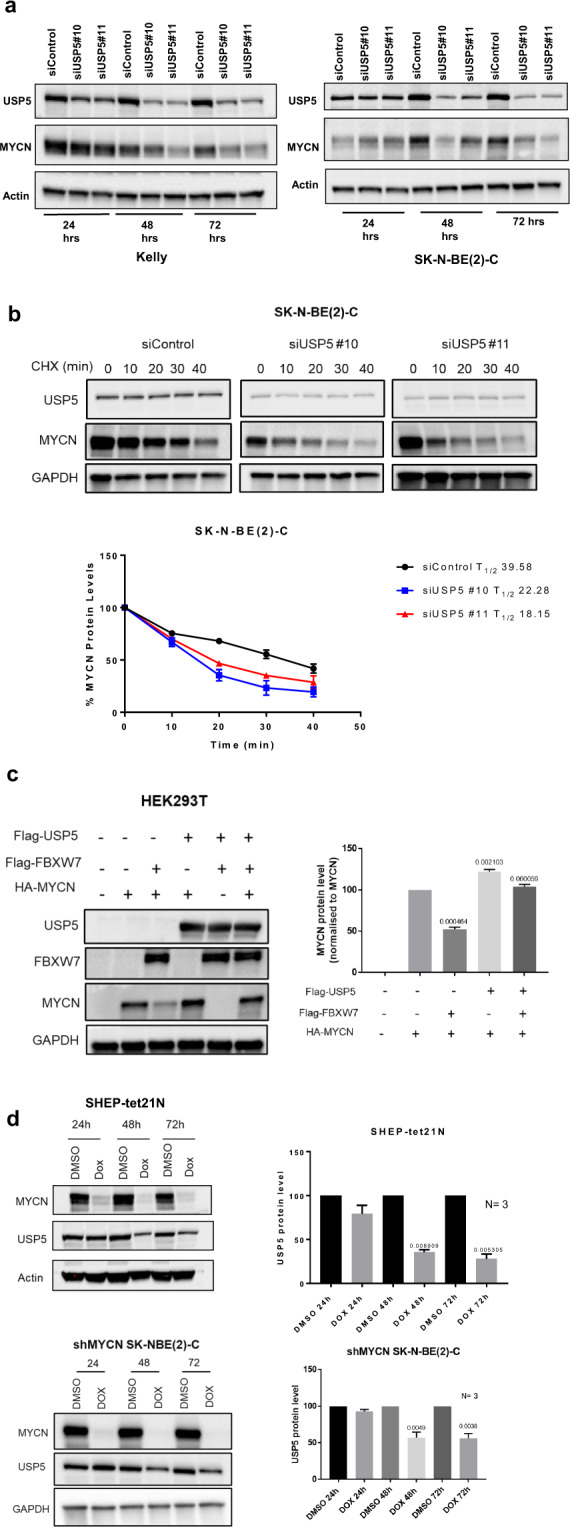

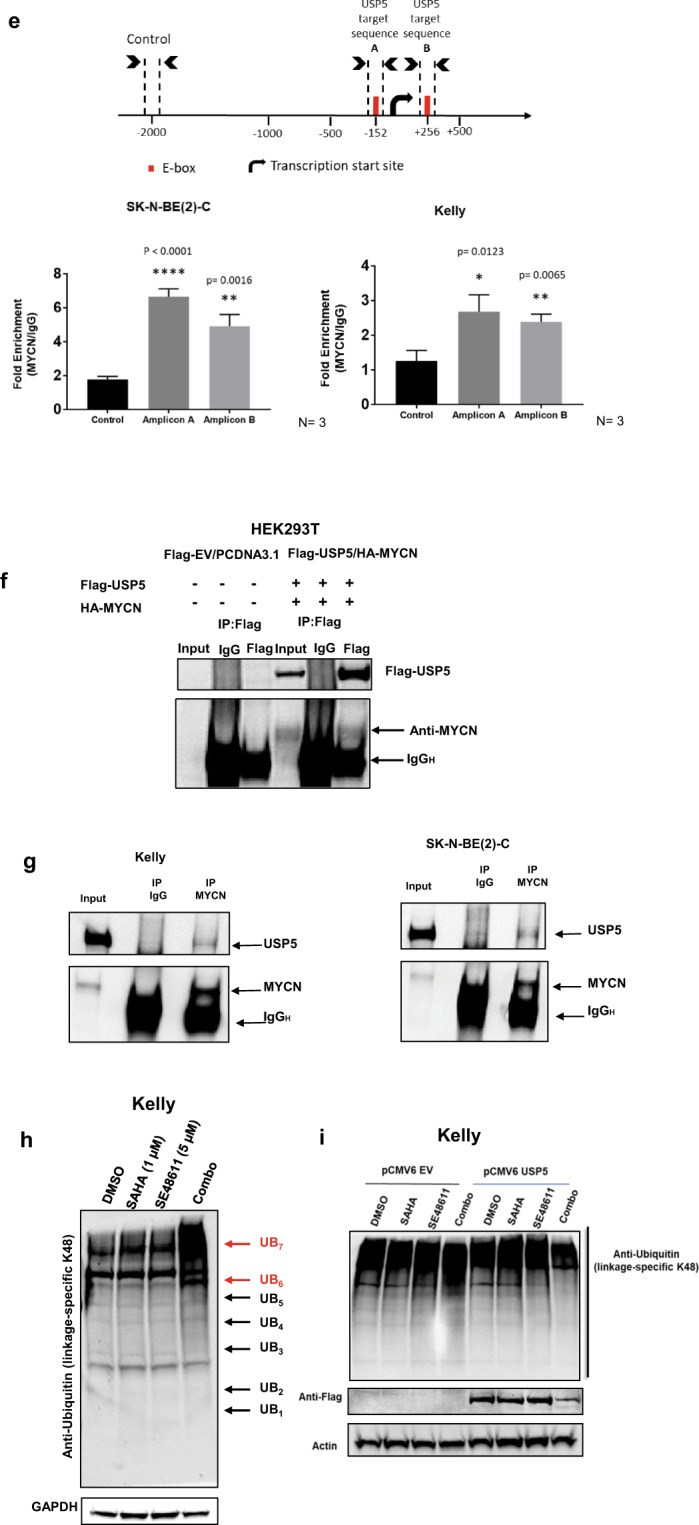
Expression of USP5 and MYCN are linked in a forward feedback loop, which can be inhibited by combination therapy effects on Lys48-specific polyubiquitin chains. **a** Kelly and SK-N-BE(2)-C neuroblastoma cell lines were transiently transfected with control siRNA or two USP5 siRNAs #10 or #11 for 24 and 48 h and immunoblot analysis was used to measure protein expression of USP5 and MYCN with USP5 and MYCN antibodies in total cell protein lysates. **b** Representative immunoblots from cycloheximide (CHX) chase assays 48 h after USP5 knockdown using control siRNA or USP5-specific siRNAs #10 or #11 in SK-N-BE(2)-C cells. Cells were then treated with 100 µg/ml CHX for up to 40 min followed by immunoblotting to detect USP5 and MYCN protein expression using USP5 and MYCN antibodies. The ratio of MYCN protein/Actin protein was artificially set at 1.0 for control samples and then half-life of MYCN protein was obtained from the line graph. **c** HEK293T cells were transfected with either control vectors, an expression vector encoding HA-tagged MYCN, Flag-tagged USP5, and/or Flag-FBXW7 as indicated. Lysates were extracted 48 h after transfection and precipitated. USP5, MYCN, and FBXW7 protein expression were detected using Flag or HA antibodies in immunoblots (left panel). Densitometric analysis was used to determine the protein expression levels of MYCN in different samples, and the *p* values were normalised against the MYCN level in lane 2 (right panel). **d** SHEP-tet21N and shMYCN SK-N-BE(2)-C cells were treated with 2 µg/ml of Doxycycline (Dox) or DMSO for 24, 48, or 72 h. Protein was subjected to immunoblot analysis of USP5, MYCN, and Actin (left panel). Densitometric analysis was used to determine the protein expression level of USP5, while Actin expression was used as a loading control (**p < 0.005 vs control) (right panel). **e** Schematic representation of the USP5 gene promoter containing the MYCN binding E-Boxs. TSS refers to the transcription start site (top panel). ChIP assays were performed with a control IgG or MYCN antibody, followed by PCR with primers targeting a remote negative control region (Control) or the E-Box regions near the TSS (Amplicon A and B) of the USP5 gene in SK-N-BE(2)-C and Kelly cells. Fold enrichment of the USP5 gene promoter region was calculated as the difference in cycle thresholds obtained with the MYCN antibody compared to the control IgG, relative to input (bottom panel). **f** HEK293T cells were transfected with constructs expressing Flag-tag-USP5, HA-MYCN, or empty vector (EV) for 24 h. Proteins from the cells were immunoprecipitated with control IgG or an anti-Flag antibody for USP5, then immunoprecipitated (IP) products were analyzed by immunoblot with a Flag or HA antibody. **g** Co-IP using IgG or MYCN antibodies of total protein from Kelly and SK-N-BE(2)-C cells, followed by immunoblotting with USP5 and MYCN antibodies. **h** Immunoblot analysis of cell protein lysates collected from Kelly cells treated for 48 h with DMSO, single agents, or combination therapy (1 µM SAHA + 5 µM SE486-11) and detection of Lys48 (K48)-linked polyubiquitin chains using Lys48-linkage-specific ubiquitin antibody. **i** Immunoblot analysis of protein lysates from Kelly neuroblastoma cells transiently transfected with either empty vector control or a USP5 expression vector for 24 h, followed by 48 h of treatment with DMSO, single agents, or combination therapy (1 µM SAHA + 5 µM SE486-11). The level of Lys48-linked polyubiquitin chains is detected using a Lys48-linkage-specific ubiquitin antibody. A Flag antibody was used to confirm overexpression of USP5 in the protein lysates.

To better understand the effect of combination therapy on MYCN protein stability and the mechanistic relationship between USP5 and MYCN, we performed immunoblotting using fractionated protein from the nuclear and cytosolic subcellular fractions of Kelly and SK-N-BE(2)-C cells. We found USP5 was located in both the nucleus and cytoplasm of MYCN-amplified neuroblastoma cells lines, whereas MYCN was predominantly located in the nucleus (Fig. S5a). We then transiently transfected Kelly and SK-N-BE(2)-C cells with two USP5-specific siRNAs. We showed that the siRNA knock-down of USP5 resulted in a significant decrease in MYCN protein levels in both cell lines after 72 h of combination treatment (Fig. 5a), and a 1.77–2.18-fold reduction in MYCN protein half-life in SK-N-BE(2)-C cells (Fig. 5b). Because MYCN is itself a target of FBXW7-mediated ubiquitination and degradation [24], we investigated whether USP5 could antagonise FBXW7-mediated MYCN degradation. Overexpression of FBXW7 for 48 h reduced MYCN protein in HEK293T cells (Fig. 5c). When USP5 was overexpressed in HEK293T cells, MYCN protein expression was increased (Lane 4). In contrast, when USP5 is overexpressed in the presence of FBXW7 and MYCN, USP5 rescued MYCN from FBXW7-mediated degradation (Fig. 5c, lane 6). Furthermore, when we used two different doxycycline (Dox)-inducible MYCN downregulation cell systems, SHEP-tet21N and shMYCN SK-N-BE(2)-C cell lines, to repress MYCN expression by treating the cells with Dox, and observed a reduction in USP5 protein and mRNA levels when MYCN was repressed (Fig. 5d; Fig. S5b). MYCN repression induced downregulation of USP5 protein was also associated with reduced cell viability in both SHEP-tet21N and shMYCN SK-N-BE(2)-C cells (Fig. S5c).

Next, we performed chromatin immunoprecipitation (ChIP) with an anti-MYCN antibody followed by real-time PCR with primers targeting a negative control region (Control), and E-boxes located −152 bp upstream of the USP5 transcription start site (Amplicon A), or an intronic region located +256 bp downstream (Amplicon B) (Fig. 5e). ChIP assays confirmed that in untreated cells the MYCN antibody immunoprecipitated the E-boxes at both Amplicon A and B at 2–6-fold higher levels than control (Fig. 5e). These findings suggest that USP5 and MYCN participate in a positive forward feedback expression loop in neuroblastoma cells, ensuring high-level expression of both proteins.

To determine whether USP5 and MYCN proteins directly bind each other, we transfected human embryonic kidney cells (HEK293T) with vectors expressing either Flag-tagged USP5 or HA-tagged MYCN, or the corresponding empty vectors. Co-immunoprecipitation of USP5 using an anti-Flag antibody then immunoblotting with a HA-specific antibody showed that MYCN and USP5 formed a protein complex (Fig. 5f). To further confirm a MYCN and USP5 interaction in a physiological setting, cell extracts from Kelly and SK-N-BE(2)-C cells were immunoprecipitated with a MYCN-specific antibody. Endogenous USP5 was detected in the MYCN immunoprecipitants from both cell lines (Fig. 5g). A major role of USP5 is regulation of the cellular levels of unanchored mono- and poly-ubiquitins [25]. USP5 can sequentially remove Lys48-linked ubiquitin from the proximal end of unanchored polyubiquitin chains in vitro and in vivo, converting unanchored polyubiquitin chains to monoubiquitins [26]. Therefore, we investigated whether the combination treatment increased the levels of Lys48-linked polyubiquitination in neuroblastoma cells through downregulation of USP5. We treated Kelly cells with either single agents or the combination, and then performed immunoblotting of total cellular protein with a Lys48-specific, ubiquitin antibody [27]. Combination treatment caused a marked increase in Lys48-linked polyubiquitin levels, compared to single agents or DMSO control (Fig. 5h). An increased ubiquitin smear was observed at Ub6 and Ub7 which is expected, since unanchored Lys-48 protein are high molecular weight proteins. Next, we transfected either the pCMV6 empty vector or pCMV6-USP5 expression vector into Kelly cells for 24 h and assessed the levels of Lys48-linkage-specific polyubiquitin chains. USP5 overexpression indeed reduced polyubiquitin levels following combination treatment, compared to the empty vector controls (Fig. 5i).

### SAHA and SE486-11 directly bind USP5

**Fig. 6:**
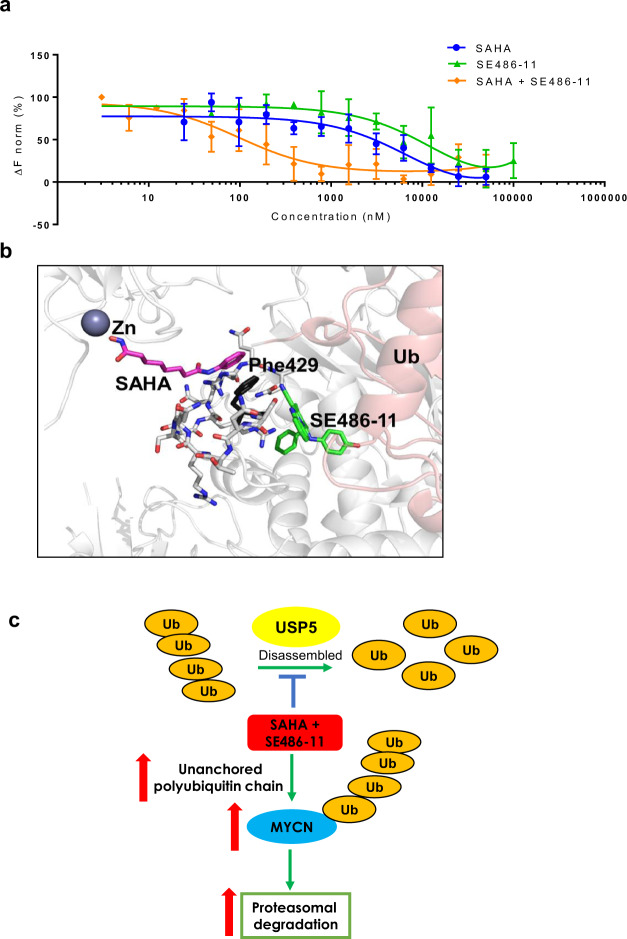
SAHA and SE486-11 bind directly to USP5. **a** Single agent and combination binding of SAHA and SE486-11 were measured using microscale thermophoresis assay and 20 µM of purified and labelled USP5 protein. When added as a single agent, SAHA (blue circles) interacted with USP5 in a dose-response manner with KD of 2.76 µM. A kinetic curve was unable to be fitted onto the SE486-11 dose-response data (green triangles). When used in combination (red squares), there was a significant increase in affinity to 159 nM. Shown is the mean ± SD of three independent experiments. **b** Hypothetical binding model of SAHA (pink sticks) and SE486-11 (green sticks) to USP5 (grey ribbon). The putative “activation loop” is shown in grey sticks, with Phe429 highlighted in purple. This residue pi-stacks with SAHA, stabilising its interaction with the zinc ion (purple sphere). A bound ubiquitin molecule is shown as a pink ribbon. **c** Hypothetic model of the combination drug inhibition and inactivation of USP5 leads to elevated unanchored polyubiquitin levels, reduced MYCN stability, and consequently lower USP5 protein expression.

To determine whether SAHA and SE486-11 could bind USP5 alone, or in combination, we performed microscale thermophoresis (MST) binding assays. SAHA was able to bind full-length USP5 in a dose-dependent manner with a KD of 2.76 µM (Fig. 6a). A kinetic curve could not be fitted to the dose-response curve of SE486-11. However, when the compounds were added in combination (a 1:5 ratio of SAHA: SE486-11 was used and showed good synergy in cell cytotoxicity assays), the binding affinity of the drug combination for USP5 was increased by more than 100-fold to a KD of 159 nM. These data suggest the compounds bind USP5 together in a synergistic fashion.

To understand the molecular basis for this binding synergy, we examined where these compounds might bind to USP5 by computational modelling. Using the known partial crystal structure of USP5 [28], in conjunction with structural and biological information from the closely related USP7 [29], we derived a molecular model of possible direct interactions between SAHA, SE486-11 and USP5. SAHA is known to bind to the active site of HDACs and acts as a chelator for zinc ions [30]. Computational modelling of SAHA binding into the zinc finger ubiquitin-binding domain (ZnF-UBP) of USP5 (residues 197–269) suggests that SAHA binds via its hydroxamate group to the zinc ion. This same SAHA group mediates the zinc chelation necessary for HDAC inhibition. Furthermore, in molecular studies of the closely related USP7, others have found that the catalytic activity was dependent on its C-terminal tail interacting with ligands in the “activation groove” [29]. However, the amino acid sequence of USP5 has a truncation in this C-terminal region, when compared to USP7. Our molecular modelling analysis using published crystal structures suggests a “stabilising loop” region (residues 423–430) could flip into this “activation groove” in USP5, acting in an analogous manner to the USP7 C-terminal tail [29]. Binding of the two drugs may cause the “stabilising loop” to flip out of the “activation groove”, leading to interference with recognition and binding of ubiquitin. By analogy with USP7, this forces the catalytic triad (Cys335, His795, Asn810) to be in a catalytically inactive form of USP5 (Fig. 6b).

The computational docking of SAHA, suggested that its benzene moiety interacts with USP5 via a pi interaction with Phe429 in the stabilising loop (Fig. 6b). Furthermore, SE486-11 was able to computationally dock into the “activation groove” of the USP5 crystal structure. Therefore, we hypothesise that SAHA binding may be synergistically enhanced by SE486-11 binding into the “activation groove”, as it would stabilise the “loop out” orientation which prevents ubiquitin binding in the activation groove of USP5.

## Discussion

Considerable evidence suggests deubiquitinases are an important therapeutic vulnerability in cancer due to their protective role in oncoprotein stability [31]. Enhanced stability of the MYCN oncoprotein is a critical driver in childhood neuroblastoma [32]. Here we show that the deubiquitinase, USP5, has an important role in maintaining MYCN stability through effects on unanchored polyubiquitin levels in neuroblastoma cells. Unexpectedly, a therapeutic combination of an HDAC inhibitor (SAHA) and a novel pyridobenzimidazole (SE486-11) had profound anti-tumour effects and synergistically bound USP5, significantly reducing both USP5 and MYCN levels (Fig. 6c). These findings have implications for therapeutic strategies aimed at lowering oncoprotein levels and the targeting of the ubiquitin proteasomal system in cancer.

Increased USP5 levels have been associated with tumorigenesis in human glioblastoma, melanoma, and hepatocellular carcinoma [33]. Indeed CRISPR/Cas-9 cancer dependency screens have recently identified neuroblastoma cells to have dependency on USP5 [34]. USP5 promotes tumorigenesis of pancreatic cancer cells by stabilising the FoxM1 protein, which is a substrate of FBXW7 [35]. Other deubiquitinases, USP28 and USP7 have been shown to increase stability of MYC [36] and MYCN, respectively [7]. Moreover, a USP7 inhibitor, P22077, reduced neuroblastoma growth through p53-mediated apoptosis [37]. Overall, these studies provide strong evidence that USPs are emerging therapeutic targets in the treatment of cancer.

We have shown that USP5 and MYCN directly interact in untreated neuroblastoma cells and, that combination therapy lowered USP5 and MYCN protein levels. The combination of SAHA + SE486-11 caused increased unanchored Lys48-linked polyubiquitin chains and MYCN ubiquitination. Moreover, these effects could be overcome by USP5 overexpression. Previous studies have shown that repression of USP5 levels caused a significant increase in unanchored polyubiquitin chains with important consequences for malignant cells, such as selective stabilisation of p53 [38]. Protein monoubiquitination is involved in the regulation of cellular processes such as DNA repair, gene expression, histone function, and receptor endocytosis [39]. Polyubiquitination, especially through ubiquitin Lys48 or Lys11, with a chain of at least 4 ubiquitins also targets some proteins for proteasomal degradation, as observed in our studies [40]. Polyubiquitination of target proteins is a kinetically favourable method for rapid degradation by the ubiquitin-proteasomal pathway. We hypothesise that this may be the favoured regulatory mechanism for oncoproteins when functioning in normal cellular processes, such as MYC and MYCN during normal cell replication, where a rapid increase and then decrease of these protein levels is required. Together, our data suggest that other overexpressed oncoprotein drivers in cancer cells, which are tightly regulated in normal cells by Lys48-linked polyubiquitination, may be vulnerable to a USP5-targeted inhibitor.

Our data indicate potent synergy between the HDAC inhibitor and SE486-11, which is explained by two mechanisms. First, we show that in neuroblastoma cells MYCN and USP5 together stimulate a forward feedback expression loop maintaining high expression of each protein. Interruption of this feedback loop would be expected to dramatically lower levels of both proteins. Second, MST shows that both drugs bind simultaneously and synergistically to USP5. We hypothesise that combination drug binding and inactivation of USP5 leads to elevated unanchored polyubiquitin levels, reduced MYCN stability, and consequently lower USP5 transcription. We cannot exclude “off target” anti-cancer effects of the combination therapy which do not involve MYCN and USP5. Indeed, the sensitivity of the SH-SY5Y cell line, which has low MYCN and USP5 expression, to combination therapy may be due to “off target” effects since sensitivity of this cell line was not caused by combination therapy reducing MYC or MYCN.

The dependence of the malignant phenotype on the presence of both MYCN and USP5 is highlighted by the marked antitumor effects of combination therapy and the low levels of both proteins in treated tumours. MYCN is largely expressed in prenatal tissues and is essential for development, making it a good target for inhibition in postnatal malignant tissues. In *Drosophila*, USP5 is also essential for development but dispensable in the adult organism, thus suggesting therapeutic inhibition of USP5 and MYCN in postnatal malignant tissues may have low toxicity to normal cells. Importantly, our in vitro experiments suggested combination therapy did not target MYC which is essential for normal postnatal cell replication. Overall, our data show that the previously described dependency of neuroblastoma cells on USP5 is due to a regulatory relationship with MYCN, which begins with effects of USP5 on unanchored Lys48-linked polyubiquitin levels. The consequent increase in MYCN protein stability is an important oncogenic driver in neuroblastoma cells. Combination therapy with an HDAC inhibitor (SAHA) and a novel pyridobenzimidazole (SE486-11) bound and inactivated USP5 with potent inhibition of tumorigenicity as a novel therapeutic approach for the treatment of MYCN-driven neuroblastoma.

## Materials and methods

### Cell lines

SK-N-AS, SK-N-FI, SK-N-BE(2)-C, SH-EP, SH-SY5Y, IMR-32, H460, A549, DU145, HCT116, HT29, PC3, SKOV3, CHP-134, Kelly, MDA-MB-231, and HEK-293T cells were purchased from ATCC by the Children’s Cancer Institute Australia Tumour Bank, and maintained in Dulbecco’s modified Eagle’s medium (Invitrogen, Australia) with 10% fetal calf serum. The human lung fibroblast cells MRC-5 and WI-38 (ATCC) were cultured in Alpha-MEM media with 10% FCS. Non-MYCN-amplified human neuroblastoma cell lines SH-EP MYCN-3 and SHEP-tet21N, which are genetically modified to overexpress or repress human MYCN cDNA when exposed to doxycycline, were generously supplied by Professor Jason Shohet (Texas Children’s Cancer Center, Houston). The shMYCN SK-N-BE(2)-C cell line are genetically modified to repress human MYCN expression by MYCN shRNA when exposed to doxycycline, and SK-N-BE(2)-C cell line was used as the parental cell line. The shMYCN SK-N-BE(2)-C cell line was derived by GenScript, Piscataway NJ. All cell lines used were authenticated by CellBank Australia (Westmead, NSW), to be free from mycoplasma, and were cultured at 37 °C with 5% CO_2_ in a humidifier incubator.

### In vivo treatment of neuroblastoma-bearing TH-MYCN homozygous mice

All experimental procedures involving mice were approved by the University of New South Wales Animal Care and Ethics Committee according to the Animal Research Act, 1985 (Australia) and the Australian Code of Practice for Care and Use of Animals for Scientific Purposes (2015). This study was approved by the Animal Care and Ethics Committee, UNSW Sydney (Approval number: 15/100B). The *TH-MYCN* transgenic mouse model of neuroblastoma used has been previously described [20]. Three-week-old female *TH-MYCN*^+/+^ mice were assigned to the four treatment groups when tumours were first palpable. The dose of SAHA was set at 35 mg/kg/day since SAHA has been widely used at that concentration in both animal studies and clinical trials, with minimal toxicity [41]. Treatment was continued for 3 weeks using a 5-days on, 2-days off schedule. Mice were humanely euthanized at the end of the 21-day period and the tumour volume (mm^3^) measured.

### Statistical analysis

All experiments included a minimum of three independent replicates. Statistical analysis was conducted using GraphPad Prism 8 software. *P* values were determined using ANOVA for multiple group comparison, or two-sided unpaired *t*-test for two groups. All values were expressed as mean values with 95% confidence intervals. Graphical error bars for represent mean ± SEM, and a *p* value < 0.05 was considered statistically significant.

## Supplementary information

Supplementary Figures and Figure Legends

Supplementary Methods
