# Supplementary material for: A novel combination therapy targeting ubiquitin-specific protease 5 in MYCN-driven neuroblastoma
Source: Oncogene. 2021 Mar 3;40(13):2367–81. doi: 10.1038/s41388-021-01712-w (PMC8016666; doi:10.1038/s41388-021-01712-w)
Supplement: Supplementary file 1 — Supplementary Figures and Figure Legends [file 41388_2021_1712_MOESM1_ESM.pdf]

Supplementary Table S1

WEHI Library Plate ID	Pilot Screen Number	Compound ID	Batch Ref	Average Percent Viability (+SAHA)	% SD (+SAHA)	Average Percent Viability (No SAHA)	% SD (No SAHA)	Difference Average Viability NoSAHA and SAHA
DE000002	1	WEHI-0006759	02	-1.25	10.61	113.04	12.89	114.30
DE000009	1	WEHI-0013384	02	14.13	3.18	126.40	12.27	112.27
DE000003	1	WEHI-0056920	01	41.06	10.15	121.99	23.98	80.93
DE000007	1	WEHI-0058053	01	-10.88	36.97	118.01	45.23	128.89
DE000007	1	WEHI-0058080	01	38.18	1.27	110.22	45.16	72.04
DE000007	1	WEHI-0058236	01	33.23	9.51	88.12	38.06	54.89
DE000009	1	WEHI-0058777	01	36.95	6.25	142.70	16.83	105.75
DE000010	2	WEHI-0059032	01	-5.52	8.96	101.50	23.49	107.02
DE000002	1	WEHI-0088362	01	2.87	3.66	74.68	17.34	71.81
DE000002	1	WEHI-0088396	01	3.93	3.31	74.00	12.30	70.07
DE000002	1	WEHI-0088486	01	27.53	16.32	85.46	9.52	57.93
DE000002	1	WEHI-0088558	01	30.45	4.37	102.59	12.89	72.14
DE000002	1	WEHI-0088568	01	43.07	23.85	115.06	16.59	72.00
DE000002	1	WEHI-0088600	01	-1.24	12.56	125.11	31.45	126.35
DE000002	1	WEHI-0088602	01	11.64	7.06	77.26	6.12	65.62
DE000002	1	WEHI-0088603	01	29.91	16.89	89.36	15.75	59.45
DE000001	1	WEHI-0088768	01	-7.21	17.72	70.01	15.96	77.22
DE000001	1	WEHI-0088846	01	37.67	12.33	99.87	16.59	62.20
DE000001	1	WEHI-0088925	01	14.41	9.86	83.89	47.07	69.48
DE000002	1	WEHI-0089012	01	-2.40	6.91	87.22	6.94	89.62
F0000001	2	WEHI-1191914	01	40.12	1.97	95.41	26.88	55.29
F0000004	2	WEHI-1193224	01	3.88	5.76	103.64	6.52	99.76
F0000007	2	WEHI-1194345	01	-21.11	9.18	73.12	29.32	94.23
F0000011	2	WEHI-1195788	01	23.80	7.49	102.88	7.10	79.09

Supplementary Figure S1

a

Type of cancer	IC50 of SAHA geometric mean (μM)
Breast invasive carcinoma	4.66
Colon and rectum adenocarcinoma	4.25
Prostate adenovarcinoma	4.60
Medulloblastoma	2.00
Neuroblastoma	2.10

Supplementary Figure S1

b

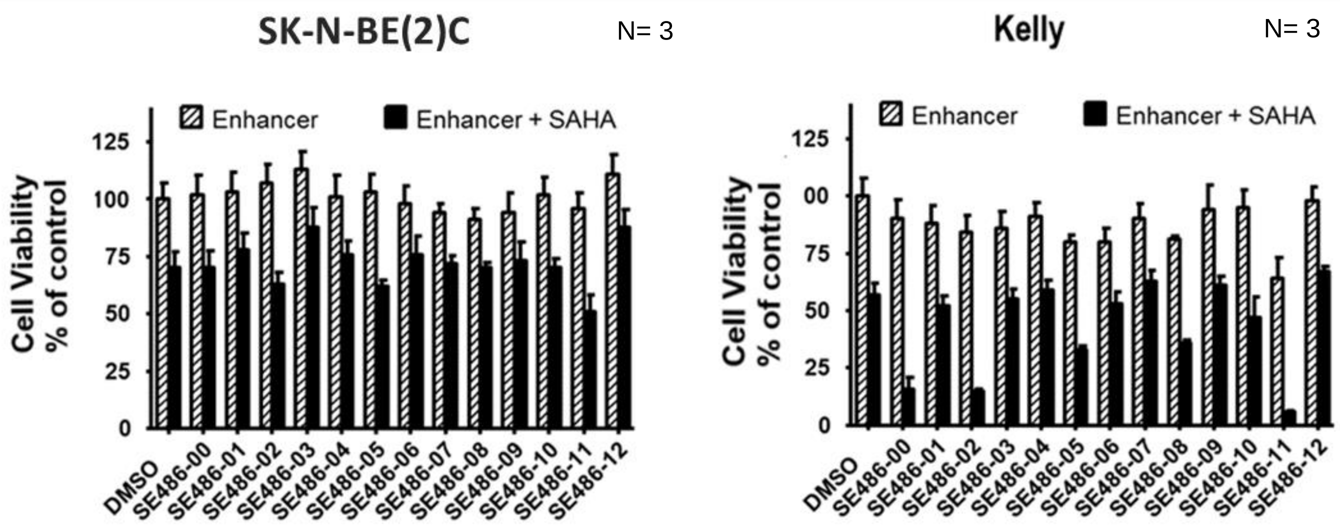

c

Cell line	Type of cancer
Kelly	Neuroblastoma
SK-N-BE(2)-C	Neuroblastoma
H460	Lung
A549	Lung
HT29	Colon
PC3	Prostrate
SKOV3	Ovarian

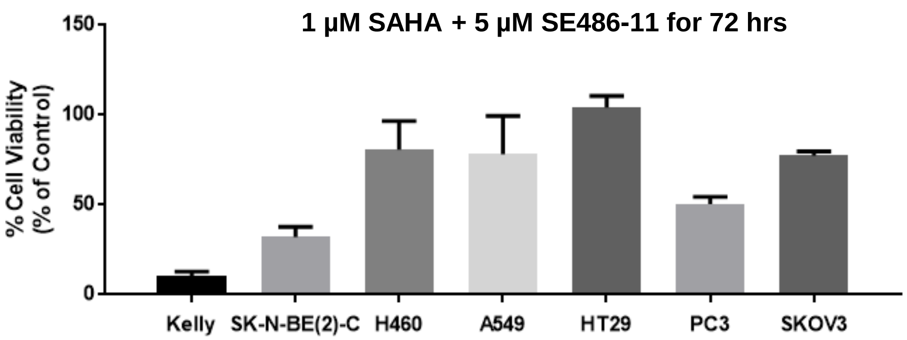

Supplementary Figure S1

d

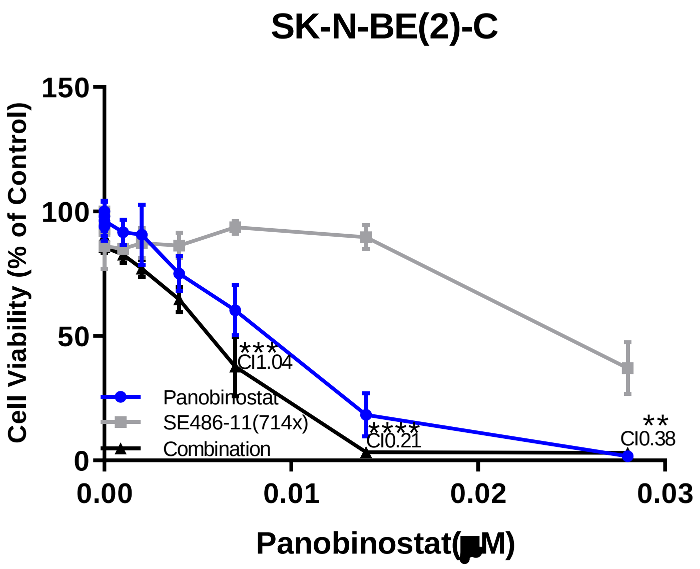

P values are SE486-11 vs combination

Summary	Adjusted P value
****	<0.0001
***	0.0007
**	0.0012

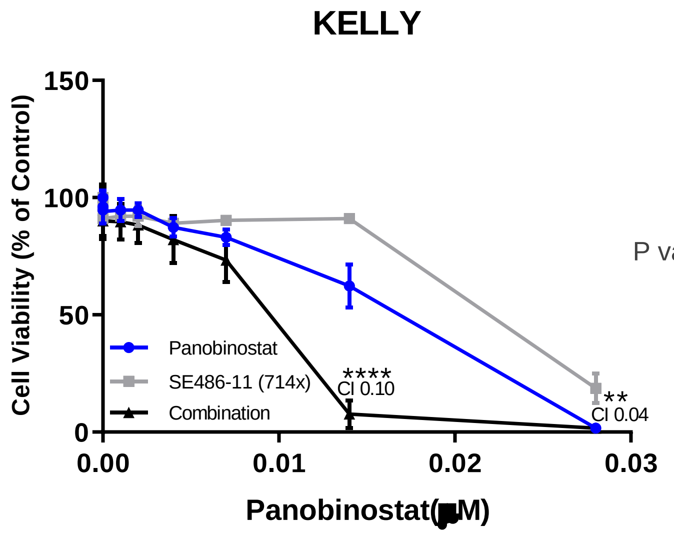

P values are SE486-11 vs combination

Summary	Adjusted P value
****	<0.0001
**	0.0037

Supplementary Figure S1

e

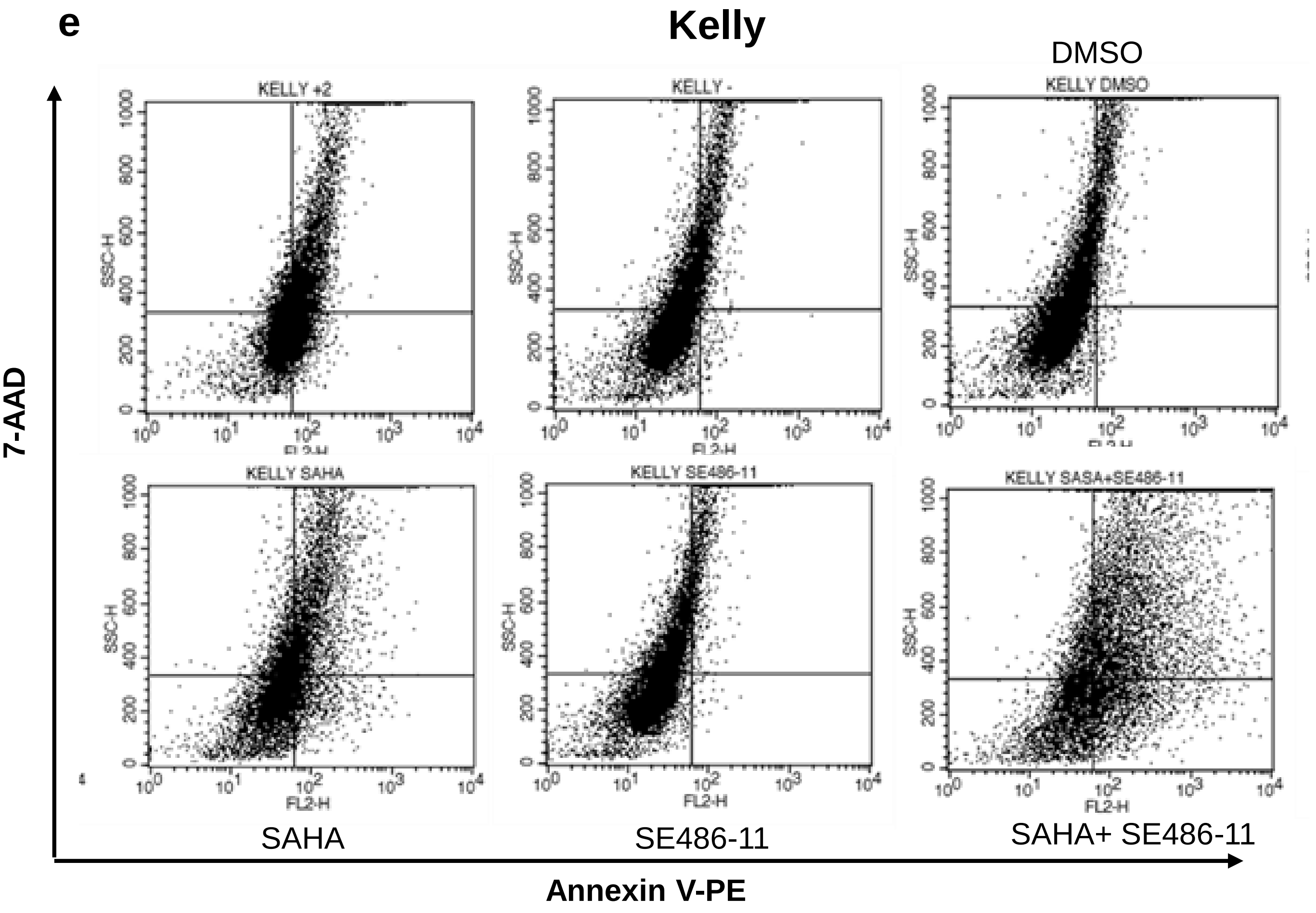

f

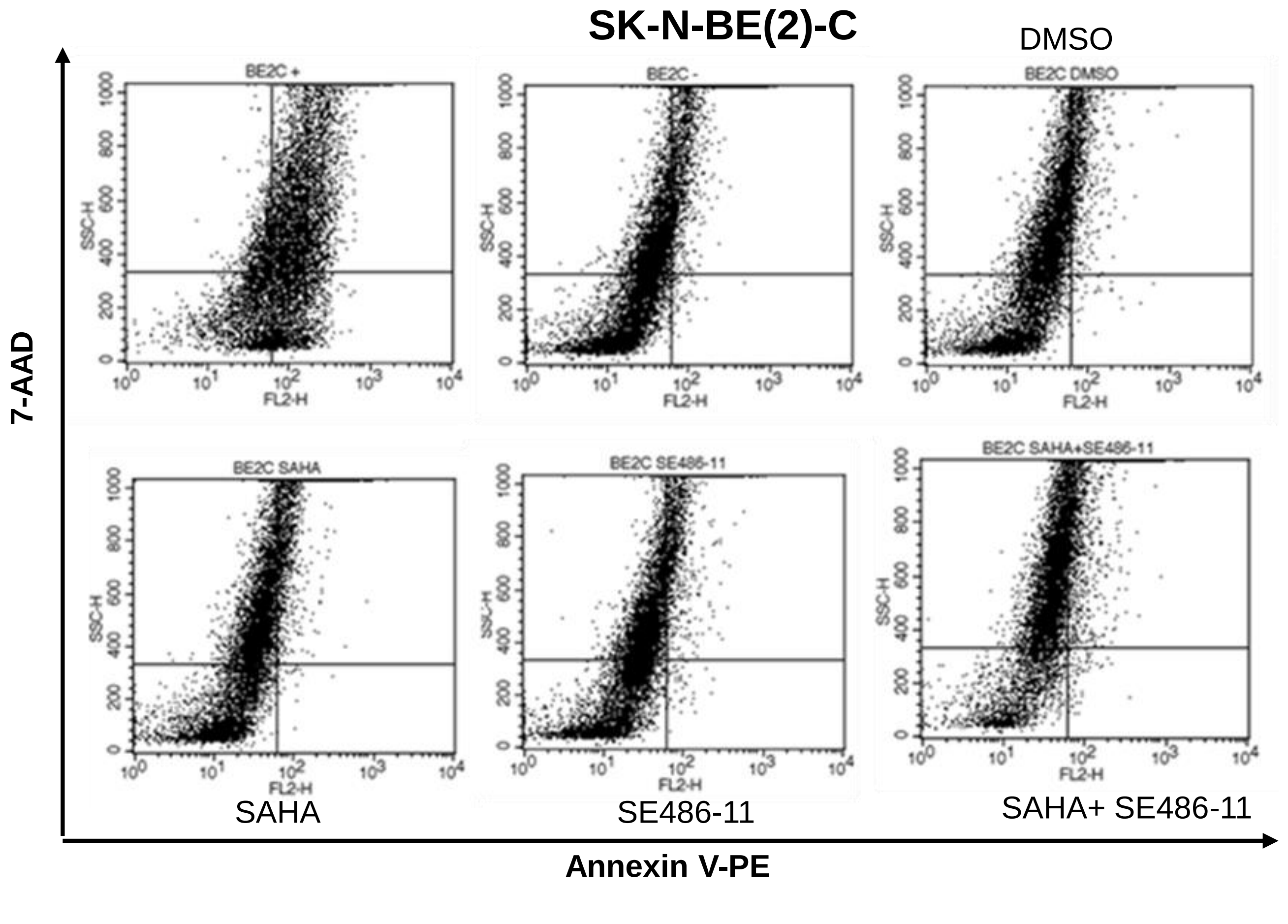

Supplementary Figure S1

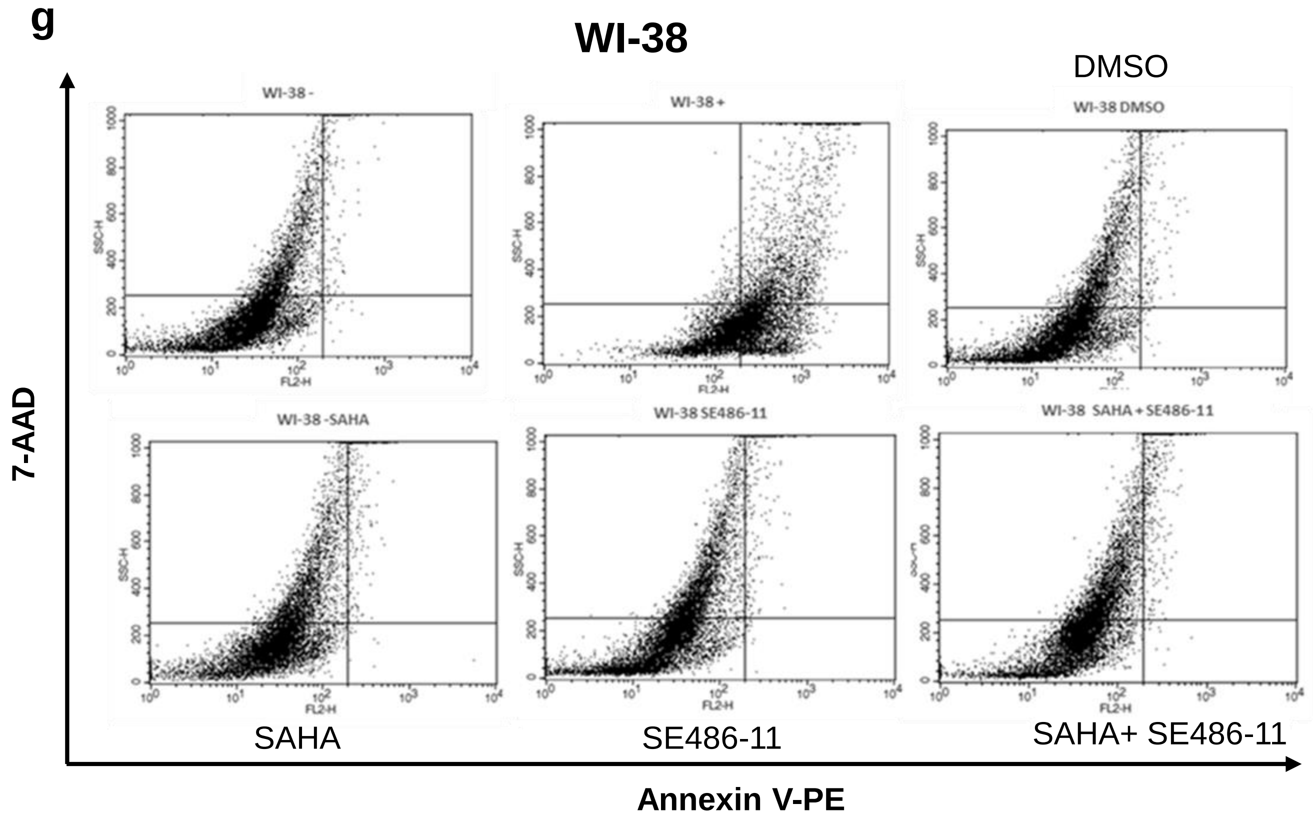

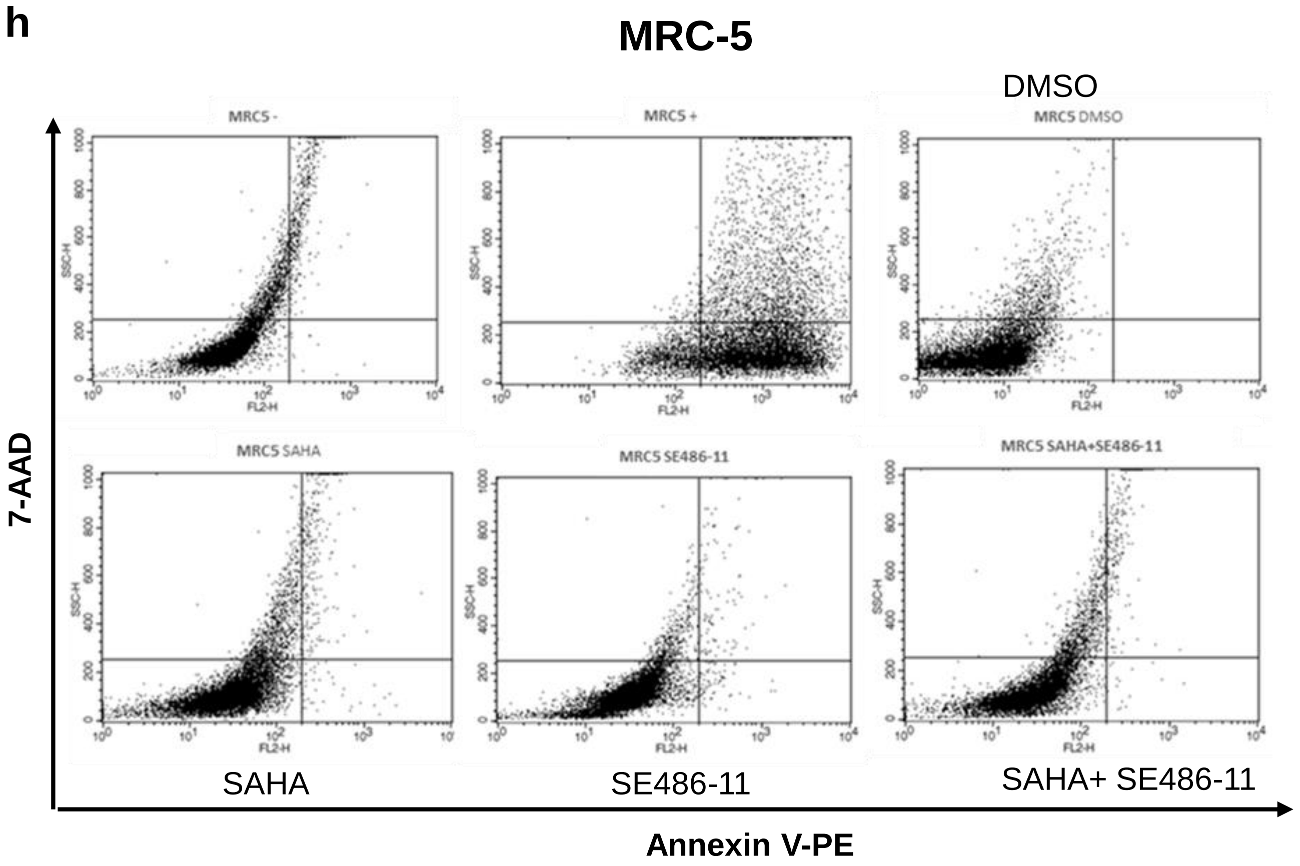

Supplementary Figure S1

i

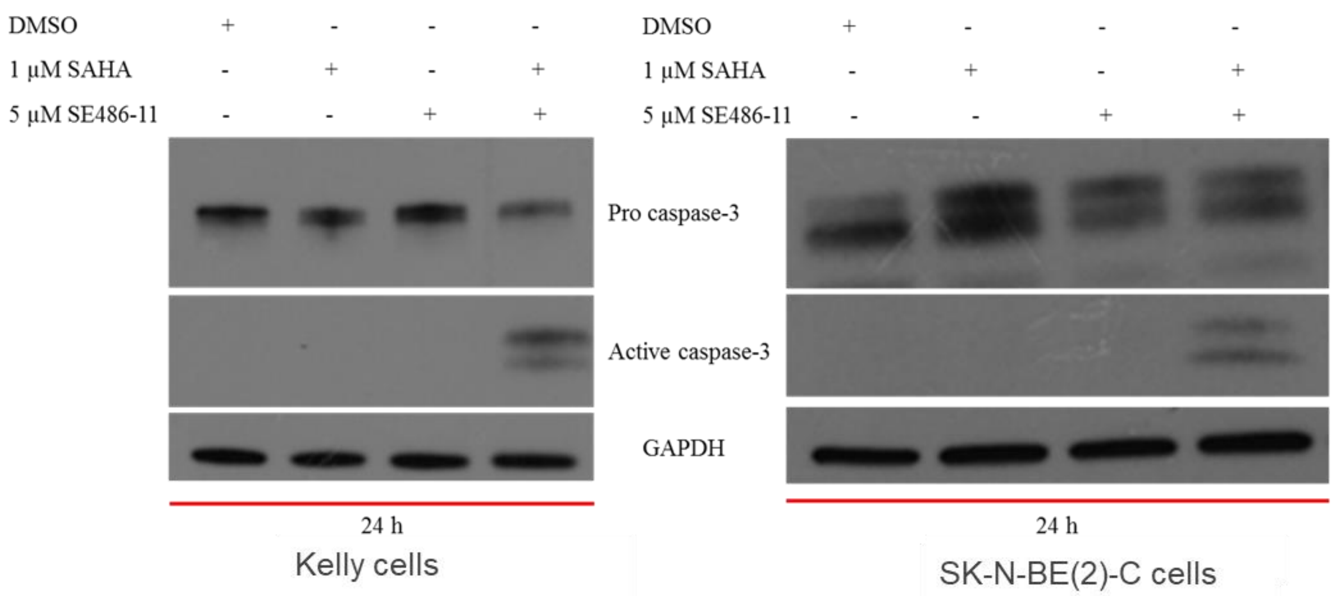

j

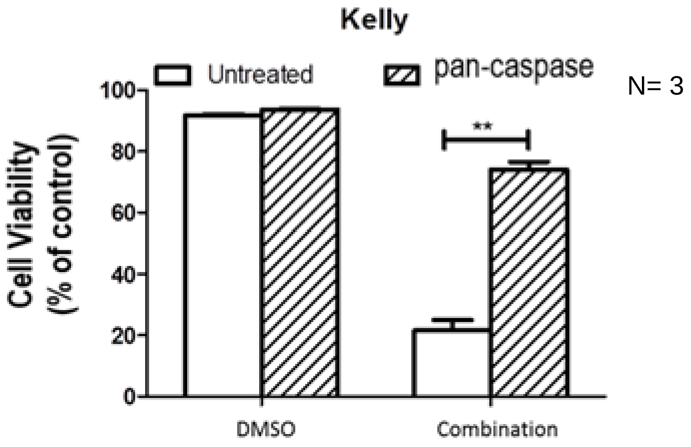

Supplementary Figure S1

k

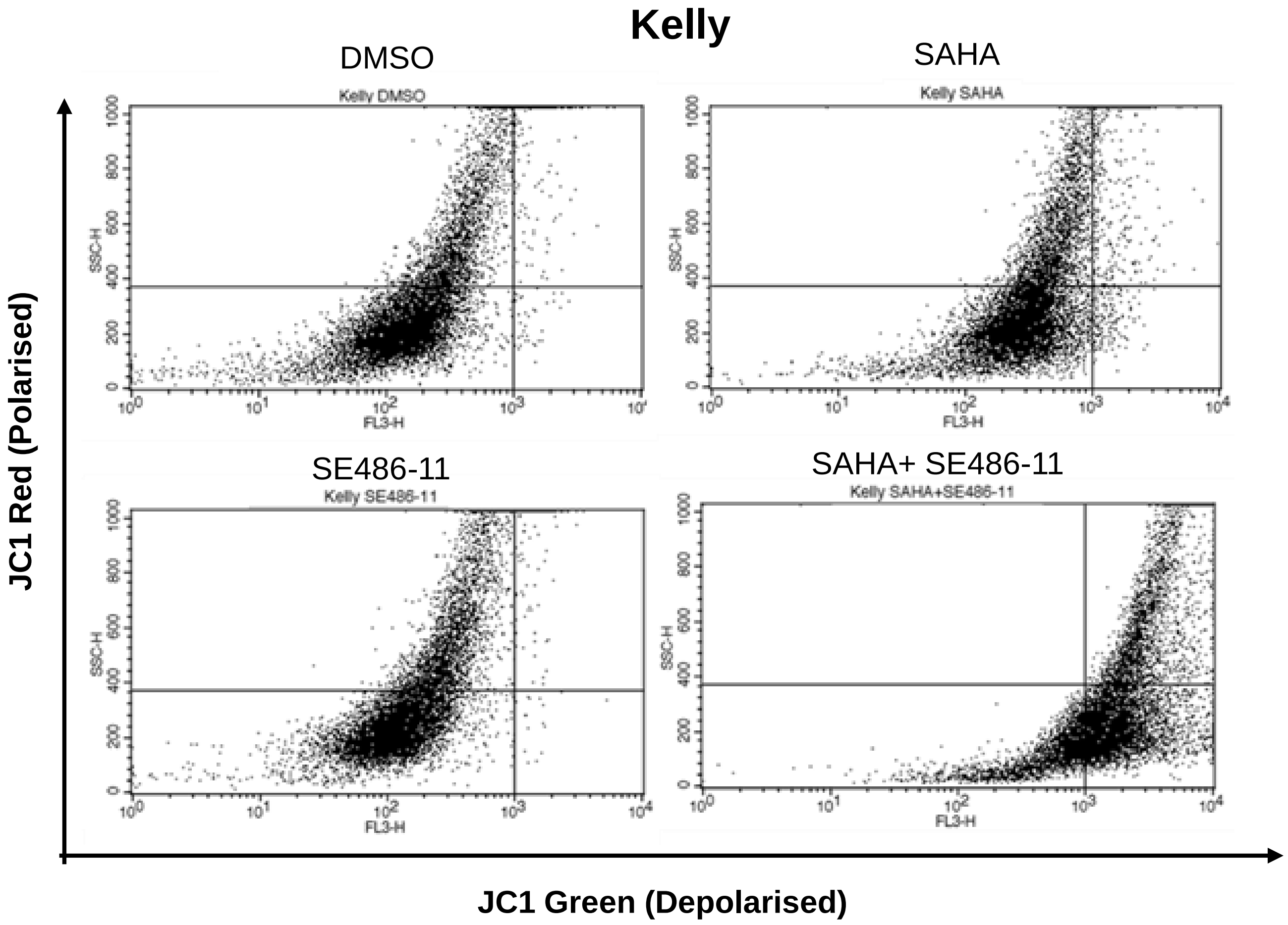

l

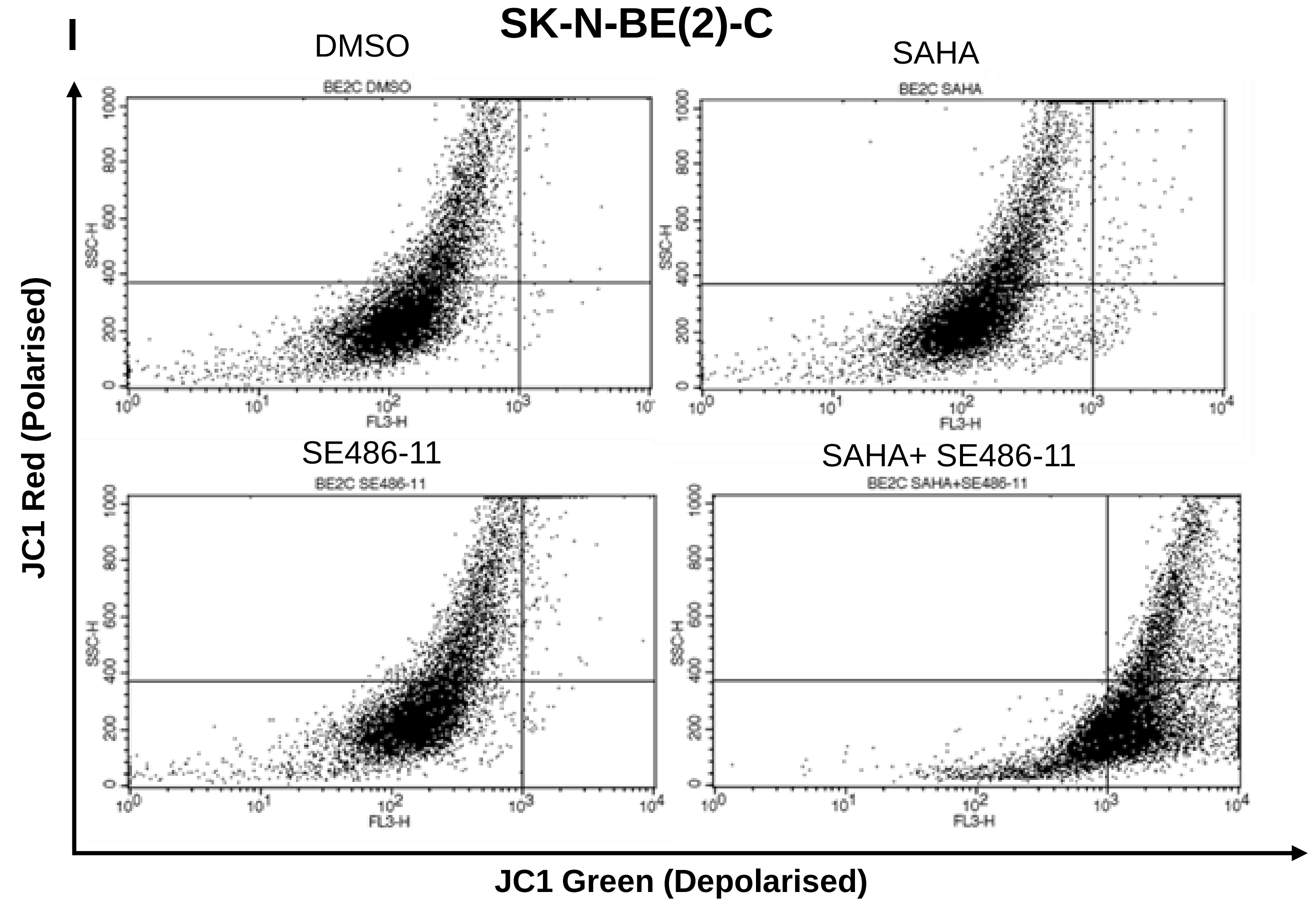

Supplementary Figure S2

a

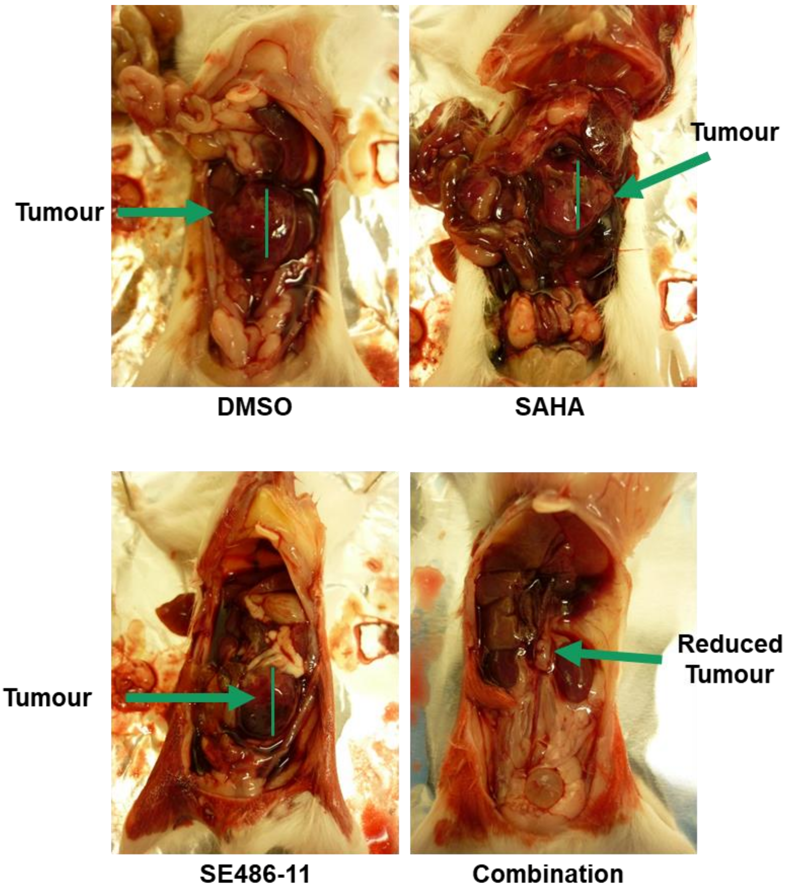

b

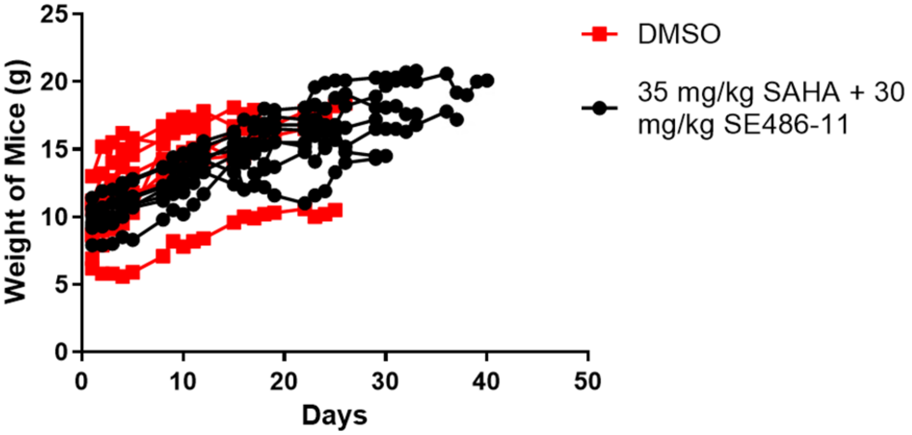

Supplementary Figure S2

c

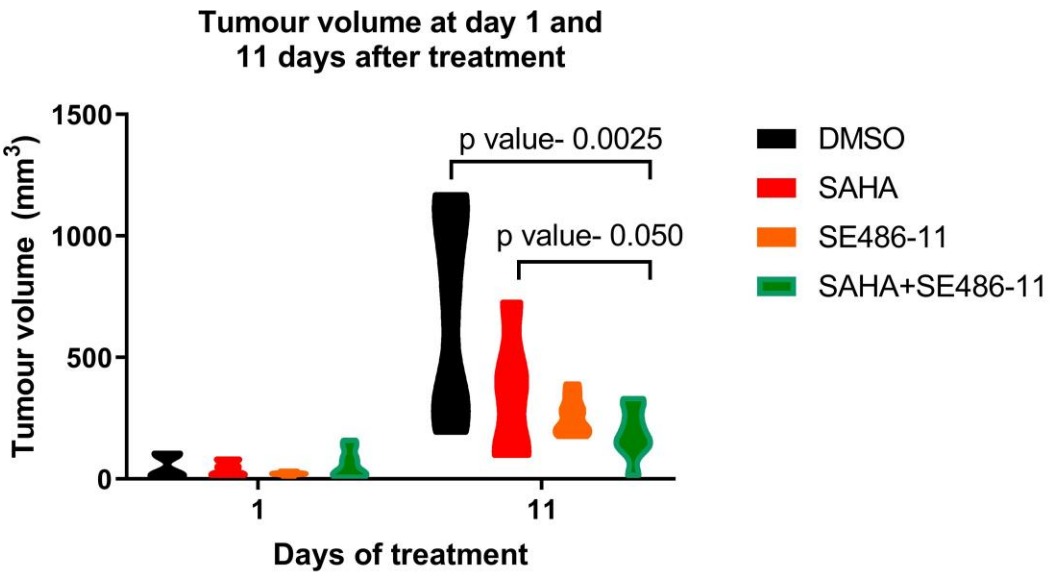

d

Representative image of tumour size at 11th day of treatment

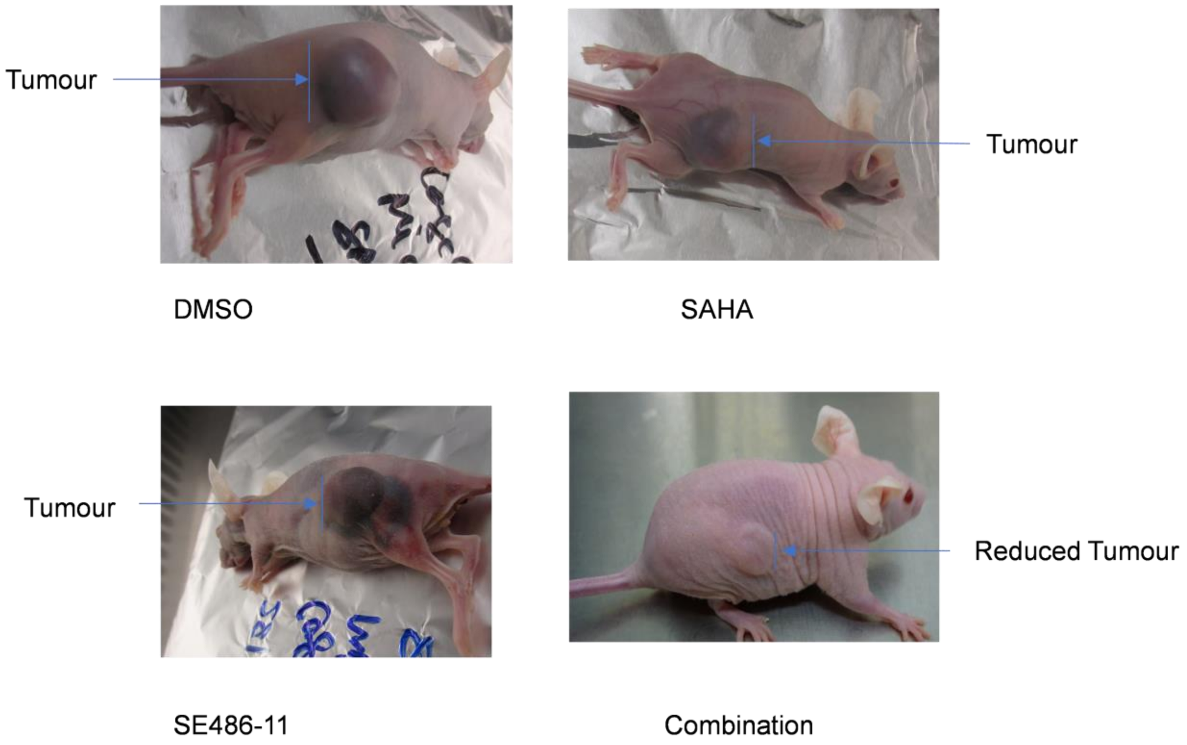

Supplementary Figure S2

e

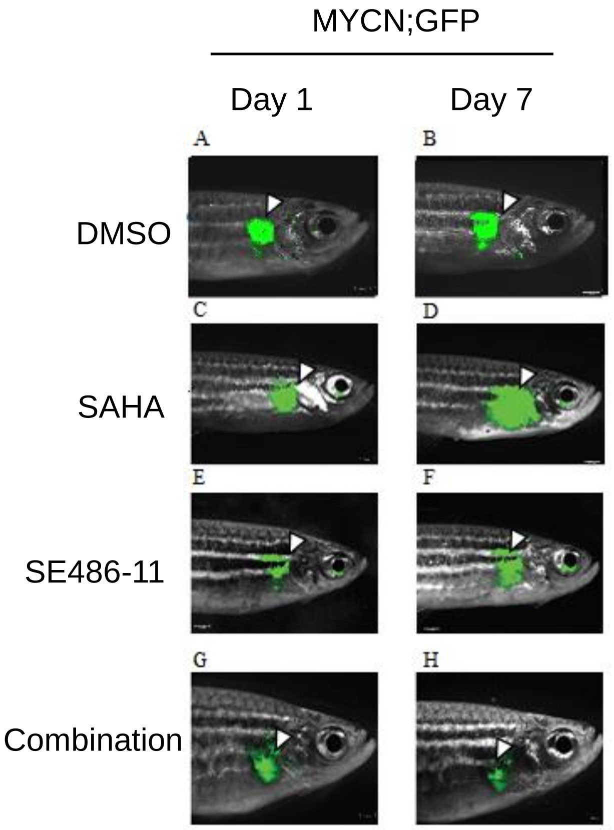

f

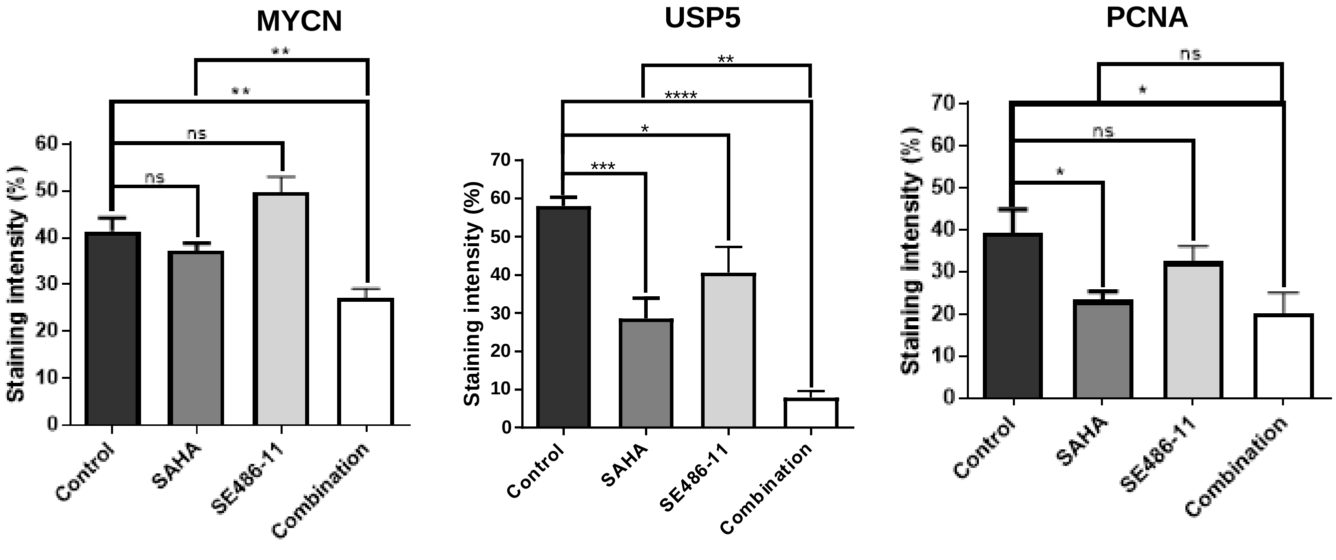

Supplementary Figure S3

a

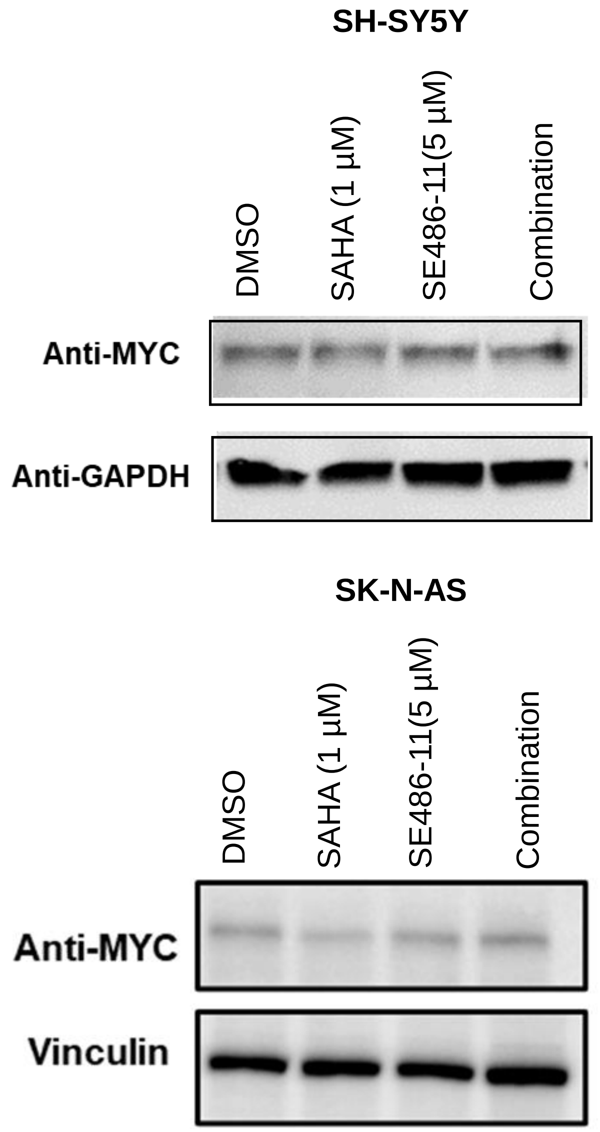

b

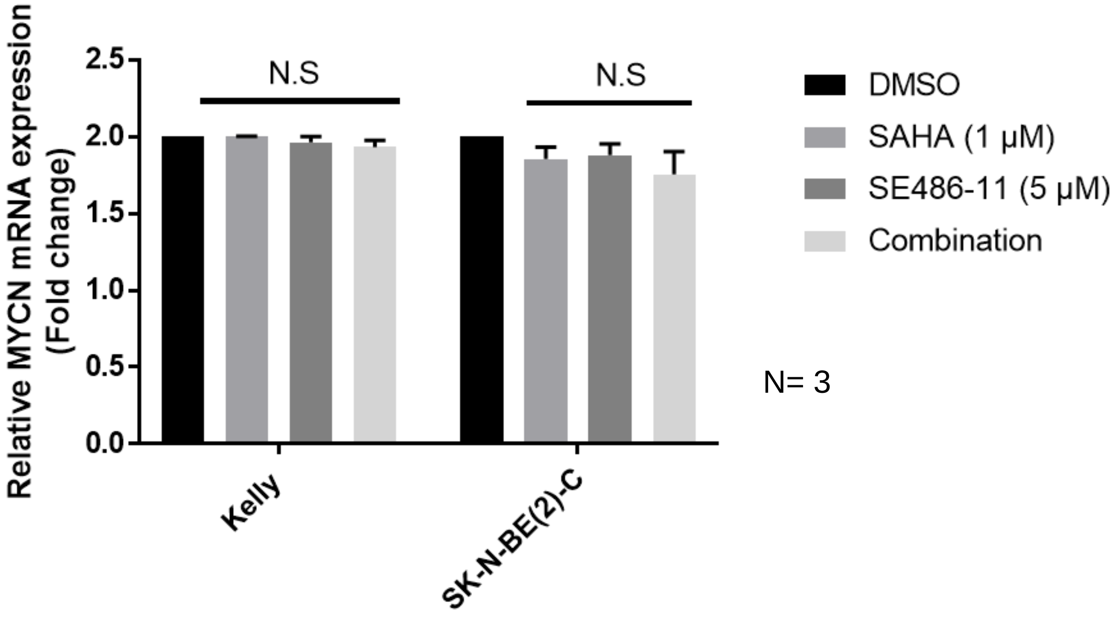

Supplementary Figure S4

a

Symbol	Name	H:L	Pval H:L
ZFR	Zinc finger RNA-binding protein OS=Homo sapiens GN=ZFR PE=1 SV=2	100	0.011766
MAK16	Protein MAK16 homolog OS=Homo sapiens GN=MAK16 PE=1 SV=2	100	0.011803
RTL1	Retrotransposon-like protein 1 OS=Homo sapiens GN=RTL1 PE=2 SV=3	1.945814	0.007455
NICA	Nicastrin OS=Homo sapiens GN=NCSTN PE=1 SV=2	1.551833	0.040031
HNRPL	Heterogeneous nuclear ribonucleoprotein L OS=Homo sapiens GN=HNRNPL PE=1 SV=2	1.352426	0.002579
TFR1	Transferrin receptor protein 1 OS=Homo sapiens GN=TFRC PE=1 SV=2	1.349654	0.009004
UBP5	Ubiquitin carboxyl-terminal hydrolase 5 OS=Homo sapiens GN=USP5 PE=1 SV=2	1.337995	0.007152
NUCL	Nucleolin OS=Homo sapiens GN=NCL PE=1 SV=3	1.316471	0.007968
MSI2H	RNA-binding protein Musashi homolog 2 OS=Homo sapiens GN=MSI2 PE=1 SV=1	1.282943	0.040135
LMNB1	Lamin-B1 OS=Homo sapiens GN=LMNB1 PE=1 SV=2	1.206284	0.038242

b

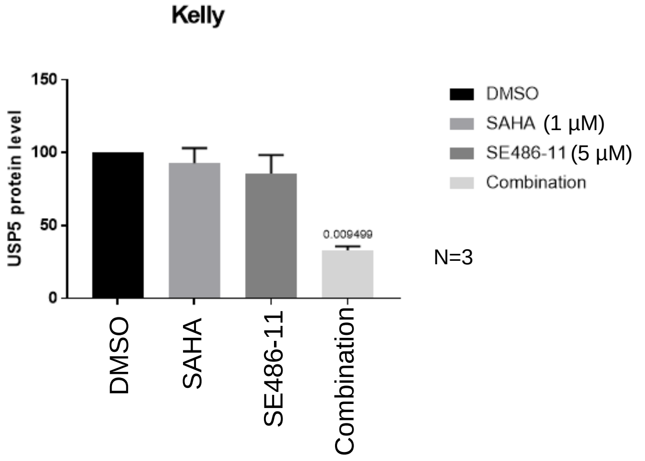

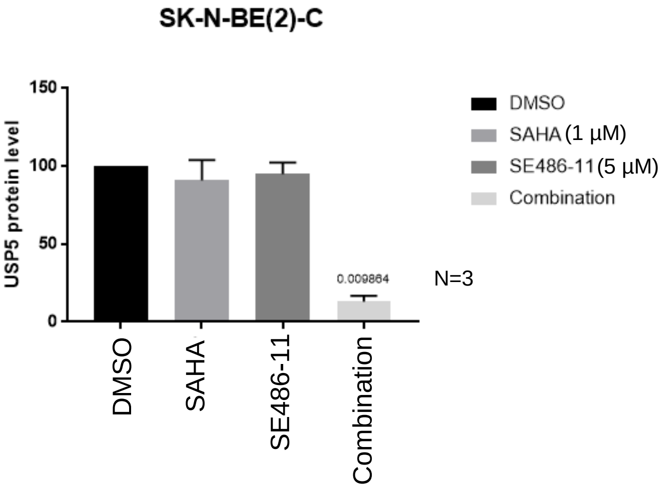

c

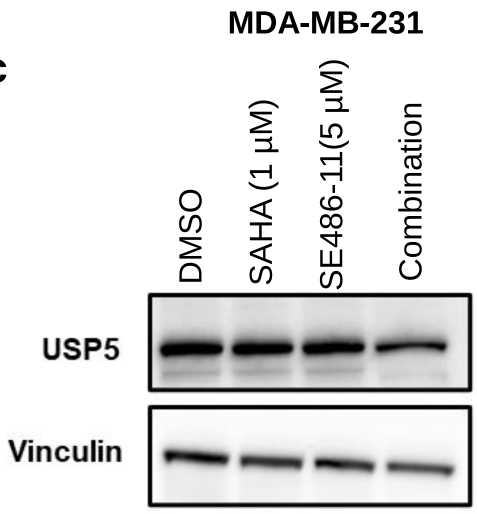

d

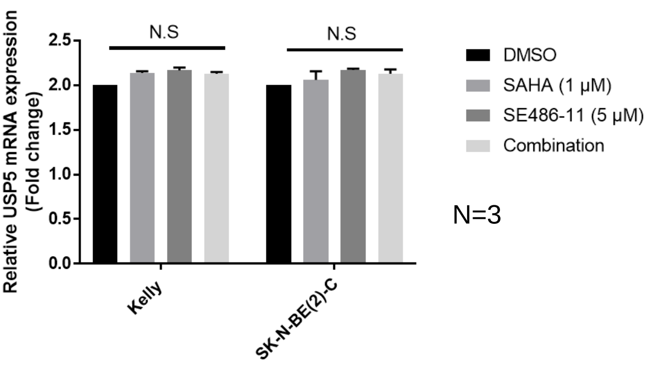

Supplementary Figure S4

e

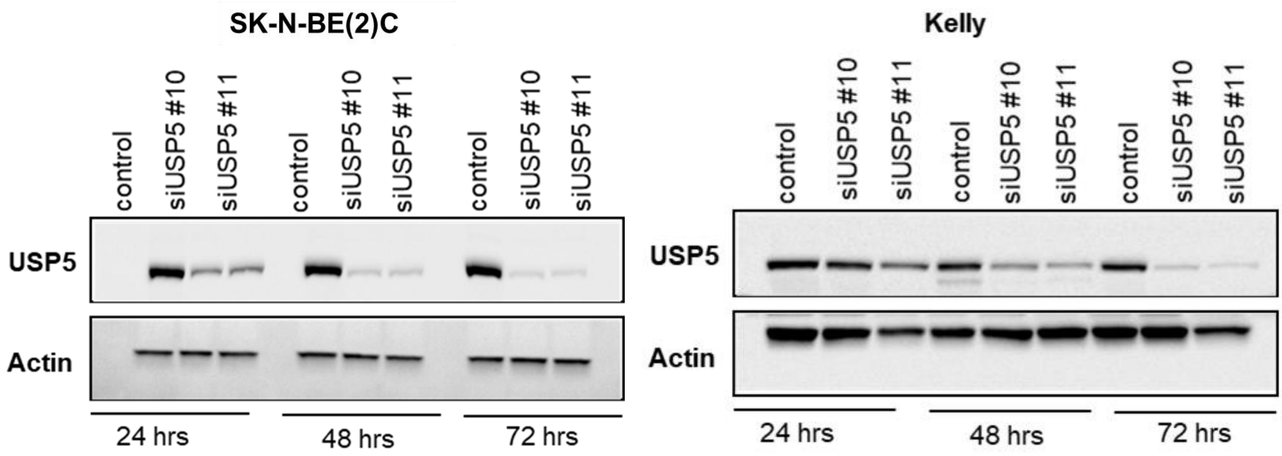

f

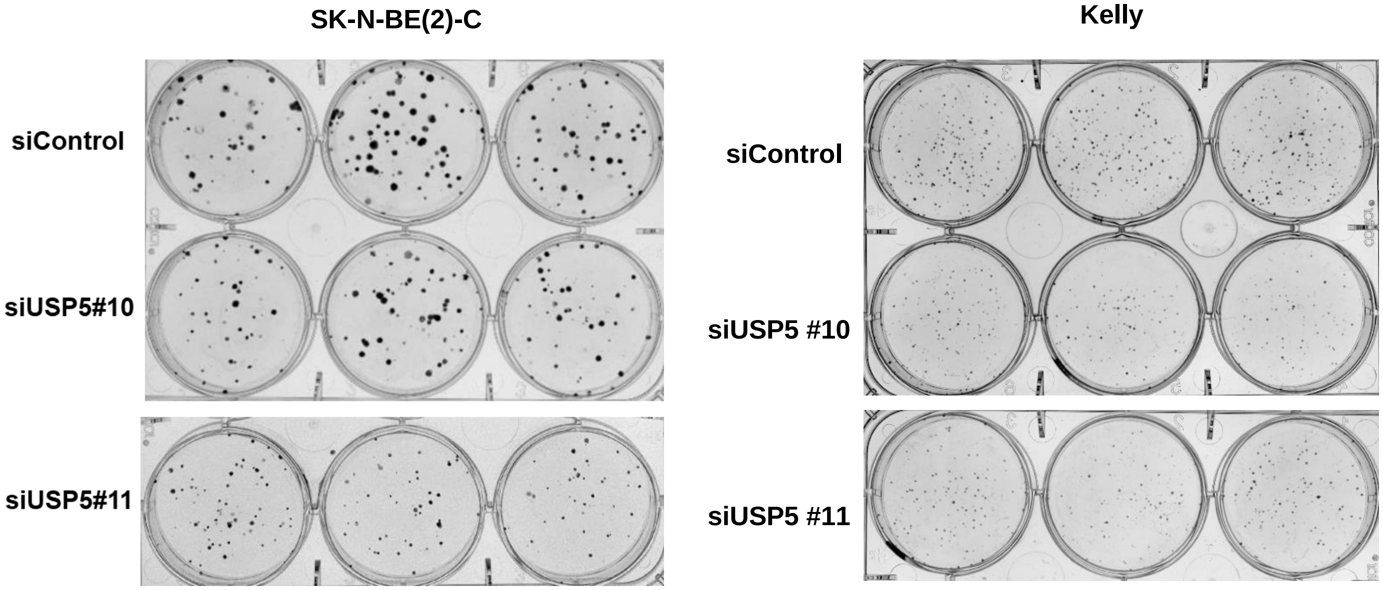

Supplementary Figure S5

a

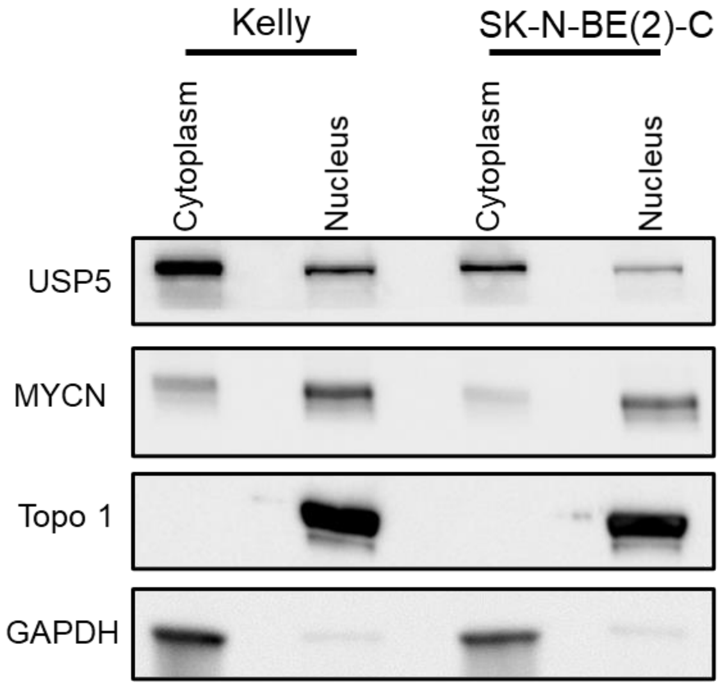

b

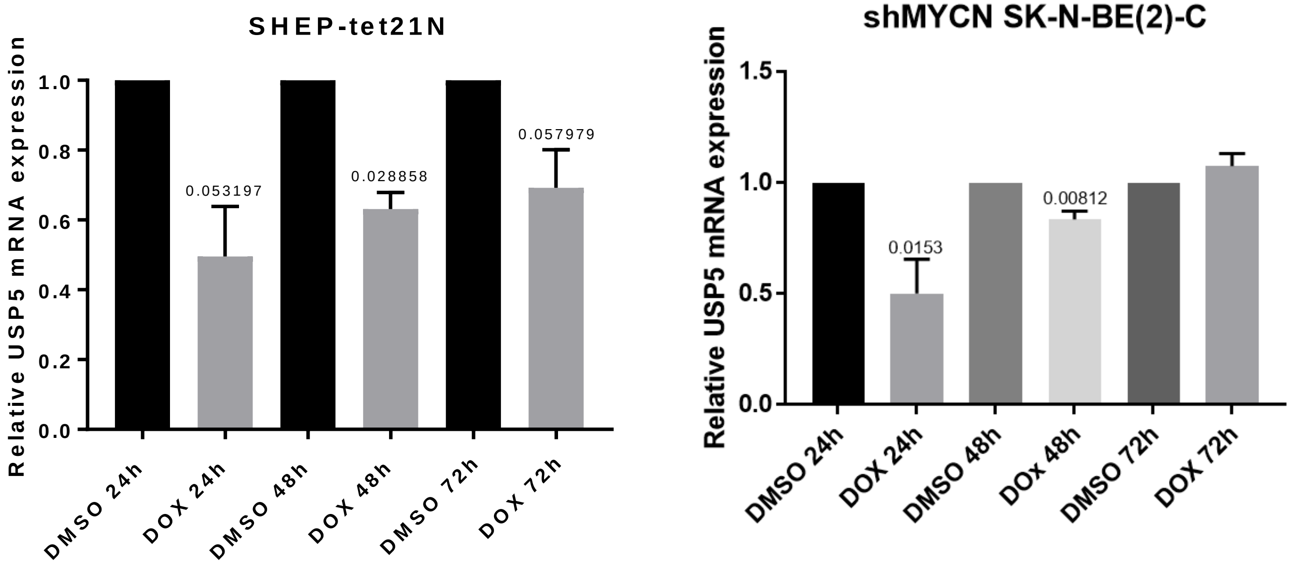

Supplementary Figure S5

c

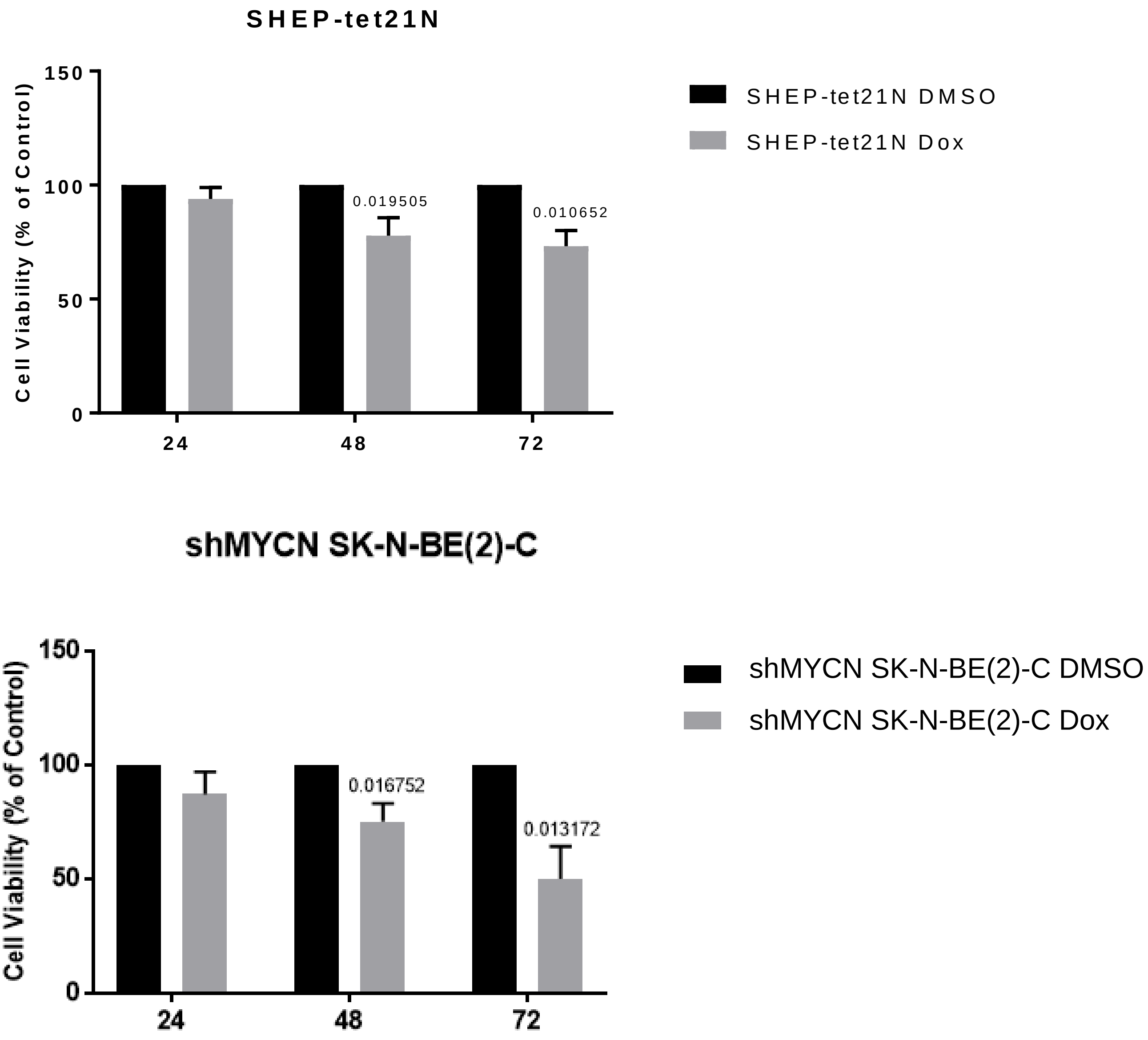

Supplementary Figure Legends

Table S1. Summary of the 24 compounds identified from the pilot and counter screens, showing greater than 40% reduction in cell viability at a compound dose of 10 µM.

Figure S1. Identification and *in vitro* evaluation of lead analogue, SE486-11, in combination with SAHA. a The Genomics of Drug Sensitivity in Cancer (GDSC) database (www.cancerRxgene.org) which was used to search information on drug sensitivity in cancer cell lines, and the drug response was used to compare the IC50 geometric mean of SAHA sensitivity across various cancer types. **b** The cell viability of single agents, or in combination with 1 µM of SAHA and 10 µM enhancers for 48 hours, as determined by Alamar Blue assay in the neuroblastoma cell lines SK-N-BE(2)-C and Kelly. **c** A panel of human cancer cell lines treated with the combination therapy for 72 hours, before measuring cell viability by Alamar Blue assay. **d** SE486-11 showed significant cytotoxicity with Panobinostat when used in combination in neuroblastoma SK-N-BE(2)-C and Kelly cell lines at different concentrations (Panobinostat : SE486-11 ratio: 1 : 714) after 72 hours treatment. Significance was determined from three independent experiments. CI <1 is synergistic. * are the adjusted p values for SE486-11 vs combination. **e-h** Representative quadrant gates for apoptosis analysis of Annexin-V/7AAD stained in neuroblastoma Kelly and SK-N-BE(2)-C cells and human normal fibroblasts, WI-38 and MRC-5 cells. The bottom left quadrant represents living cells, the cells in the top left quadrant represents necrotic, the top right quadrant represents late apoptosis and the bottom right quadrant represents cells undergoing early apoptosis. **i** Immunoblot analysis of Kelly and SK-

N-BE(2)-C cells pre-treated with DMSO, single agents, or combination therapy for 24 hours before collection of cell protein lysates and detection of pro-caspase 3 and active-caspase 3 using a caspase 3 antibody. **j** Cell viability measured by Alamar Blue assay in Kelly cells following 48 hours treatment with DMSO or combination therapy (1 µM SAHA + 5 µM SE486-11), and the pan-caspase inhibitor, Q-VD-OPh. **k-l** Representative gating/analysis of JC-1 stained Kelly and SK-N-BE(2)-C cell lines. JC-1 assays representing the proportion of cells with depolarised mitochondria in each of the two cell lines after 48 hours of the treatment with DMSO, SAHA, SE486-11 or the combination.

Figure S2. *In vivo* tumour growth in transgenic *TH-MYCN*$^{+/+}$ homozygous mice and MYCN;GFP zebrafish after combination therapy. a The representative images of *TH-MYCN*$^{+/+}$ mice at the end of 3 weeks of treatment with single agents or combination therapy. **b** *TH-MYCN*$^{+/+}$ mice were treated with DMSO or combination therapy from age 3 weeks for a period of 21 days using a 5 day on and 2 days off schedule and their weight was measured twice weekly. **c** Tumour volumes were measured at day 1 and day 11 post-treatment of SK-N-BE(2)-C xenograft mouse models with SAHA, SE486-11 or the combination and displayed here as violin plots. **d** Representative images of tumour sizes at 11 days of the treatment. **e** Representative immunofluorescence images of EGFP-expressing tumours (arrowhead) in the MYCN;GFP transgenic zebrafish were treated with vehicle, single agents, or the combination for 7 days by oral gavage. **f** Differences in staining intensities in sagittal sectioned-stained slides of neuroblastoma tumours from MYCN;GFP zebrafish treated with DMSO, SAHA, SE486-11 and combination. Graphs illustrate the percentage of the histogram intensity measured by Image J software for

H&E, MYCN, USP5 and PCNA expression. Sample means (horizontal bars) were compared by ANOVA for multiple group comparisons. *represents p-value <0.05. ns represents p-value of no significance.

Figure S3. Combination therapy effects on MYC expression in SH-SY5Y neuroblastoma cells. a SH-SY5Y and SK-N-AS neuroblastoma cell lines were treated with either DMSO, single agents, or combination therapy (1 µM SAHA + 5 µM SE486-11) for 48 hours. Cell protein lysates were collected, and MYC protein expression were detected using a c-MYC antibody. **b** Real-time PCR analysis of MYCN mRNA expression following treatment of DMSO, 1 µM SAHA, 5 µM SE486-11 or combination for 48 hours in Kelly and SK-N-BE(2)-C cells. The difference in MYCN mRNA expression between control, single agents or combination are statistically non-significant.

Figure S4. USP5 is the only ubiquitin-specific protease downregulated following combination treatment and promotes cell growth of neuroblastoma cells. a List of the top 10 proteins downregulated following 48 hours of treatment with combination therapy versus control, as measured using the quantitative proteomics SILAC assay. "H : L" stands for Heavy labelled cells (H, the cells treated with the SAHA + SE486-11 combination) versus Light unlabelled cells (L, the cells treated with DMSO). The H : L column compares the peak intensities of the heavy and light peptides to determine the changes in protein expression of the proteins identified by mass spectrometry. "Pval H:L" represents the significance level for changes in protein expression after combination therapy. This was done by comparing the expression of the protein when cells were treated with DMSO versus when cells were treated with combination. **b**

Kelly and SK-N-BE(2)-C cell lines were treated with either DMSO, single agents, or combination therapy for 24 and 48 hours. Densitometric quantification of immunoblotted USP5 protein expression in Kelly and SK-N-BE(2)-C cells using USP5 and GAPDH antibodies. *represents p-value <0.05. ** represents p-value <0.01. **c** MDA-MB-231 cells were treated with either DMSO, single agents, or combination therapy for 48 hours. Cell protein lysates were collected, and representative immunoblots of USP5 expression detected using a USP5 antibody are displayed. **d** Real-time PCR analysis of USP5 mRNA expression following DMSO, 1 µM SAHA, 5 µM SE486-11 or combination treatment of Kelly and SK-N-BE(2)-C cells for 48 hours. The difference in USP5 mRNA expression between control, single agents or combination was statistically non-significant. **e** Immunoblots from SK-N-BE(2)-C and Kelly neuroblastoma cell lines transfected with two USP5 siRNAs (siRNA#10 and #11) for 24, 48, and 72 hours. Proteins from the cells were probed with USP5 or actin antibodies. **f** Three replicates plates of stained colonies for SK-N-BE(2)-C and Kelly cell lines 24 hours after transient transfection with either control siRNA or two USP5 siRNAs (#10 and #11), followed by 14 days of growth.

Figure S5. MYCN regulates USP5 mRNA transcription and suppression of MYCN reduces cell viability. a Immunoblotting showing USP5 and MYCN expression in MYCN amplified neuroblastoma cells using fractionated protein from the nuclear (N) and cytosolic (C) subcellular fractions. USP5 and MYCN protein expression were detected using USP5 and MYCN antibodies. Topoisomerase 1 (Topo 1) and GAPDH antibodies were used as loading controls in the nuclear and the cytoplasmic compartments, respectively. **b** Real-time PCR analysis of USP5 mRNA expression in SHEP-tet21N and shMYCN Sk-N-BE(2)-C cells following the treatment of 2 µg/ml of

Doxycycline (Dox) or DMSO for 24, 48 or 72 hours. Densitometric analysis was used to determine the mRNA expression of USP5 and used β2 microglobulin as control (**p<0.005 vs control). **c** Cell viability measured by Alamar Blue assay in SHEPtet21N and shMYCN SK-N-BE(2)-C cells following the treatment of 2 µg/ml Dox for 24, 48 and 72 hours.
