# Supplementary material for: A novel combination therapy targeting ubiquitin-specific protease 5 in MYCN-driven neuroblastoma
Source: Oncogene. 2021 Mar 3;40(13):2367–81. doi: 10.1038/s41388-021-01712-w (PMC8016666; doi:10.1038/s41388-021-01712-w)
Supplement: Supplementary file 2 — Supplementary Methods [file 41388_2021_1712_MOESM2_ESM.docx]

**Supplementary Methods**

**High-throughput drug screen and derivation of SE486-11**

*r*

An automated 384-well cell-based viability assay for identifying compounds that synergise with SAHA was performed on MDA-MB-231 cells. The Multidrop 384 (Thermo Scientific; PathTech) was used to seed the assay plates with specific cell numbers and to add CellTitre-BlueTM viability reagent to cells. The Caliper Sciclone ALH3000 liquid handling system was used for the addition of 100 nl of 1.9 µM SAHA, positive and negative controls and compounds to 50 µl of cells (and media) to produce a final DMSO concentration of 0.1%. Twister II arms and associated stackers were used to deliver plates to and from the deck of the liquid handler. The Wallac EnVision 2100 plate reader (Perkin Elmer) was used to measure fluorescence at λ ex 535 nm / λ em 590 nm at the time of addition of CellTitre-BlueTM viability reagent (time 0) and at 5 hours (h) after incubation at 37^o^C. Fluorescence signals were acquired using an optimised resorufin detection label. A Minitrak™ Robotic Liquid Handling system (Perkin Elmer) was used to titrate compound “hits” using DMSO. A Reach-In CO_2_ Incubator (ThermoForma) was used to maintain assay plates at 37^o^C prior to the addition of 1.9 µM SAHA, positive and negative controls and compounds, and during the 5h incubation period with CellTitre-BlueTM viability reagent. An Envision™ Multilabel Plate Reader (Perkin Elmer) was used for absorbance measurements. The addition of plate stackers allowed automated reads of consecutive plates. The ActivityBase (ID Business Solutions or IDBS) suite of software was used to analyse the percent cell viability.

The WEHI (Walter and Eliza Hall Institute of Medical Research) compound library consists of >100,000 compounds, each dissolved in neat DMSO to yield a final compound concentration of 5 mM. In the pilot screen 10,560 compounds were randomly selected for testing from the D and F copies of the library. A total of 100 nl was transferred to give a final concentration of 10 µM. The D copy was designed to have at least 75% of the compounds display “lead-likeness” by using a series of functional group filters, similarity filters and drug-like filters. In general, the compounds were below 400 in molecular weight, had 1 to 4 ring structures, < 5H bond donors and < 8H bond acceptors. In design of the F copy of the library, 15,000 compounds were identified and purchased based on re-analysis of frequent hitter sub-structures found from numerous previous screens using the D copy. Compounds were made up in Matrical 384-well 50µl V-bottomed plates. Compound plates were sealed for overnight storage using foil seals from Beckman Coulter. Hits were cherry-picked from hit picking plates stored at 15^o^C and deposited in Matrical 384-well 50 µl V-bottomed plates. Based on the chemical structure of one of the “hit” compounds, SE486, we searched the Hit2Lead (ChemBridge) website and identified 12 compounds as a focused library (SE486-01 to SE486-12), which had more than 80% structure similarity with SE486. SE486-11 was selected for further study.

**Cell viability and proliferation assay**

Cells were seeded in 96 well plates overnight at densities to achieve > 90% confluency. SAHA was purchased from Cayman Chemical (Michigan, USA. Catalog number; 10009929). SE486-11 was purchased from ChemBridge (San Diego, CA. Catalog number; SC-5870103). Panobinostat was purchased from Thermo Fisher Scientific Australia. To assess drug cytotoxicity, cells were treated with the compounds of interest for a period of 24, 48, and 72 hours prior to being analyzed using the Alamar Blue assay (Invitrogen). To assess cytotoxicity in the absence, or with overexpression, of a protein of interest, cells were either transfected with USP5 (pCMV6-USP5 DNA plasmid) or MYCN (HA-MYCN) for a period of 24 hours (for overexpression) or with USP5 siRNA#10, USP5 siRNA#11, or siControl (Dharmacon) for a period of 24, 48, or 72 hours (for knockdown of USP5). The Alamar Blue assay was used to measure the cell’s metabolic activity and colorimetric change (fluorescence) using the Victor 3 Spectrophotometer (Perkin Elmer) at 560nm excitation and 590 nm emission. To assess cell proliferation, BrdU incorporation was measured using the Cell Proliferation BrdU kit (Sigma, NSW, Australia) according to manufacturer’s instructions. Changes in cell proliferation were calculated from the absorbance readings at 370 nm (495 nm reference wavelength) on the Benchmark Plus microplate reader (Bio-Rad).

**Colony formation assays**

Cells were transfected using USP5 siRNA#10, USP5 siRNA#11, or siControl (Dharmacon) for a period of 24 hours. Kelly cells and SK-N-BE(2)-C cells were seeded into 6-well plates at a density of 400 or 200 cells per well, respectively. To assess drug cytotoxicity, cells were seeded and pre-treated for a period of 24, 48 or 72 hours. After the treatment period, media (10% FCS DMEM) was replaced every third day and colonies were left to grow for 14 days until visible colonies appeared. Cell colonies were stained using crystal violet (0.5% (w/v) and quantified using image J.

***In vivo* treatment of neuroblastoma xenografts**

Human neuroblastoma cell line SK-N-BE(2)-C at a cell count of 3 million cells were suspended in 0.2 ml of serum-free media and injected subcutaneously, using a 25 gauge needle, in the dorsal flank of BALB/c-nude mice of 4-5 weeks of age. When the mice had a detectable tumour 4-5 mm in diameter, they were randomized to controls (DMSO), SAHA, SE486-11, and SAHA+SE486-11 groups. Mice were treated by i.p. injection 5 days per week, with 2 days off, for up to 6 weeks or tumour size reached above 1000 mm^3^. Tumour volume was measured using digital calipers twice a week and when tumour size reached > 1000 mm^3^, mice were humanely killed and the tumours were excised for molecular analysis.

**Western immunoblotting and co-immunoprecipitation assays**

For immunoblotting, cells were harvested, and the pellets lysed with RIPA buffer containing protease inhibitors (Sigma-Aldrich). For co-immunoprecipitation assays, cells were harvested, and the pellet was lysed in ice-cold BC100 buffer (20 mm Tris-HCl, pH 7.9, 100 mm NaCl, 10% glycerol, 0.2 mm EDTA, 0.2% Triton X-100, and freshly supplemented protease inhibitor) and incubation on ice for 30 minutes. After centrifugation at 12,000 x g for 20 mins at 4°C, and the supernatant was collected. For immunoblotting, 20-40 µg (calculated using the ThermoFischer Scientific BCA Assay kit) of whole protein lysates were resolved on either 10.5% or 10-14% Tris-HCl Criterion gels (Bio-Rad, NSW, Australia). MYCN and GAPDH antibodies were purchased from Santa Cruz Biotechnology. USP5 and K48-linkage-specific ubiquitin antibodies were purchased from Abcam. The Flag antibody was purchased from Cell Signaling, and mouse IgG and rabbit IgG antibodies were obtained from Sigma-Aldrich. The HA-tagged MYCN vector was kindly provided by Professor Wei Gu (Columbia University, New York). The Flag-USP5 and FBXW7 vectors were purchased from OriGene Technologies (Rockville, MD). For co-immunoprecipitation immunoblots, 750 µg of cell lysate was incubated with either MYCN, Flag, or control mouse IgG antibody, then captured by Gammabind G-Sepharose beads (GE Healthcare). Bound proteins were resolved by SDS-PAGE. All primary antibodies were probed overnight at 4^o^C and secondary antibody incubation was carried out at room temperature for 2-4 hours. Immunoblots were visualized after incubation with Clarity™ Western ECL substrate and imaged with the ChemiDoc-Touch Imaging System (Bio-Rad, NSW, Australia). Densitometry of protein expression was measured using Image Lab software (Bio-Rad) and each protein expression band was normalized to the GAPDH loading control. For the cellular localization of USP5 immunoblotting experiments, Kelly and SK-N-BE(2)-C cells were fractionated into cytoplasmic and nucleus compartment using the Thermo Scientific NE-PER Nuclear and Cytoplasmic Extraction Kit and method described in the kit. Thirty µg of cell protein lysates were collected, and protein was quantified using immunoblotting.

***In vivo* treatment of neuroblastoma-bearing MYCN-transgenic zebrafish and immunohistochemistry**

All zebrafish were maintained at the Mayo Clinic Zebrafish Facility (MCZF; Rochester, MN) established in 2007. Therapeutic procedures were approved by the Institutional Animal Care and Use Committee (American Association for Laboratory Animal Science) according to the Humane Care and Use of Animals Policy (2015), and the Health Research Extension Act of 1985. Transgenic zebrafish with established neuroblastomas were treated via oral gavage, once per day with the following regimens: i) 2μl of vehicle (DMSO); ii) 1μl of SAHA (21 mg/kg); iii) 1μl of SE486-11 (30 mg/kg), or iv) combination of 1μl of SAHA (21 mg/kg) plus 1μl of SE486-11 (30 mg/kg). After 7 days of treatment, fish were anesthetised and sacrificed for sectioning and pathology analyses. Bright-field images of H&E or immunostained paraffin sections (3-5 µm each) were captured by an Olympus BX53 compound microscope equipped with an Olympus DP71 camera. Images were processed with Zeiss Zen 2012, Adobe Photoshop and Illustrator CS3 (Adobe) software. The acquired H&E or immunostained images were quantified by measuring the intensity of stained tumour cells using ImageJ software. The mean of changes in staining intensity between the four treatment groups were compared by ANOVA for multiple group comparisons (GraphPad prism 8).

**SILAC protein labelling and quantitative proteomics analysis**

SK-N-BE(2)-C cells were grown in DMEM with 10% FCS and either ‘light’ [^12^C_6_]-L-lysine or ‘heavy’ [^13^C_6_]-L-lysine SILAC media for over six generations to achieve >97% labelling efficiency. Cells were then pre-treated with DMSO or combination treatment (SAHA 1 µM + SE486-11 5 µM) and allowed to grow for a period of 48 hours before being harvested. The cell lysates of the ‘light’ and ‘heavy’ labelled cells were mixed 1:1 and proteins were separated by SDS-PAGE. Using the In-Gel tryptic digestion kit MS-compatible peptides were generated. The peptides were cleaned with C18 ZipTips (Millipore) according to the manufacturer’s instructions, followed by analysis with LC-MS/MS. The resulting MS/ MS data were processed using MaxQuant with integrated Andromeda search engine. The peak intensities of the heavy and light peptides were compared to determine the changes in protein expression of the identified proteins with combination treatment. The comparative significance of the fold changes in protein expression were done by calculating P values. The P values were calculated by comparing the expression of the proteins from the cells treated with DMSO versus the cells treated with the combination.

**Measuring mRNA using an inducible MYCN system**

SH-EP Tet21/N and shMYCN SK-N-BE(2)-C inducible MYCN overexpression cells were plated at a density of 5X10^5^ per T75 flask and treated with 2µg/mL of Dimethyl sulfoxide (DMSO) (Sigma, NSW, Australia) or Doxycycline (Dox) for 24, 48 or 72 hours. Total RNA was harvested using Purelink RNA mini kit (Invitrogen) according to the manufacturer’s protocol. cDNA was synthesized using the CDNA synthesis kit (Bioline) according to the manufacturer’s protocol. qPCR analysis was conducted on the Applied Biosystems Veriti 96-Well Thermo Cycler using Power SYBR Green PCR Master Mix (Applied Biosystems). Validated primers specific to each target gene was used. Primer sequences used are listed below (Integrated DNA Technology and Sigma):

| USP5 | Forward | GAGGCGCTGCTGTCAGTATT |
| --- | --- | --- |
| USP5 | Reverse | GTTTCCCAAAGCCCAGAAAC |
| MYCN | Forward | CGACCACAAGGCCCTCAGTA |
| MYCN | Reverse | CAGCCTTGGTGTTGGAGGAG |
| beta 2 microglobulin | Forward | ACTGGTCTTTCTATCTCTTGTA |
| beta 2 microglobulin | Reverse | TGATGCTGCCTTACATGTCTCG |

**Cellular apoptosis and mitochondrial depolarization**

Neuroblastoma cell lines were seeded in T75 flasks to achieve 90% confluency after 24 hours, and pre-treated with SAHA (1 µM), SE486-11 (5 µM), the SAHA + SE486-11 combination (1 µM + 5 µM) or DMSO for 48 hours before harvesting. Apoptotic and necrotic effects of the single agents or combination treatment was determined by staining the cells with Annexin V PE/7-AAD using the Annexin V: PE Apoptosis Detection Kit (BD Pharmigen, NSW, Australia) as per the manufacturer’s instruction. The mitochondrial depolarisation of the collected cells, following pre-treatment, was determined by the JC-1 assay performed using the MitoProbe™ JC-1 Assay Kit for Flow Cytometry (Life Technologies, NSW, Australia) as per the manufacturer’s instructions. Samples were analyzed by flow cytometry using the FACSCalibur (BD Biosciences, Macquarie Park, NSW, Australia) and the data analyzed using the FlowJo software (Ashland, OR, USA).

**Chromatin immunoprecipitation (ChIP)**

ChIP assays were performed as per manufacturer’s instructions using a ChIP Assay kit (Merck, Millipore) with mouse anti-MYCN antibody (Santa Cruz Biotech) and mouse control IgG antibody (Invitrogen). Real-time PCR analyses of ChIP assay products were performed using primers designed to cover the promoter regions of the USP5 gene containing Myc responsive E-Box sequences, or remote negative control regions. Fold enrichment of the USP5 gene promoter regions by the anti-MYCN antibody was calculated by dividing cycle threshold values of PCR products from the USP5 gene promoter regions by cycle threshold values of PCR products from the negative control region, relative to input. Sequences of primers used are listed as following:

| USP5 promoter | Forward | TGGGATTGGTTCTTTCAACC |
| --- | --- | --- |
| USP5 promoter | Reverse | AATGAGCGCCACTCGTTAG |
| USP5 intron 1 | Forward | CGTTGCCGTTCTAAGCTAGG |
| USP5 intron 1 | Reverse | GAGAACAGTGGGGAGTCGTC |
| Negative control | Forward | CCATGGACCTTATCCCAAAA |
| Negative control | Reverse | CCAAGGAGGAAGCAAGACAG |

**Proteasomal inhibition**

Cells were treated with combination therapy (SAHA 1 µM + SE486-11 5 µM) for 24 hours and then treated with 30 µM of the proteasome inhibitor, MG-132 (Biomol, USA) for 4 hours. Cells were harvested and the expression of MYCN analyzed by western immunoblotting using the anti-MYCN antibody.

**Cycloheximide Chase Assays**

SK-N-BE(2)-C cells were seeded at a density of 250,000 cells/well in a 6-well plate before transfection using USP5 siRNA#10, USP5 siRNA#11, or siControl (Dharmacon) for a period of 48 hours. Transfected cells were treated with 100 µg/ml of cycloheximide (Sigma, NSW, Australia) for 0, 15, 30, 45 or 60 minutes. Cells were washed with cold PBS after treatment and harvested. The cell pellet was resuspended in 200 µl RIPA buffer containing protease inhibitor (Sigma-Aldrich, NSW, Australia) and centrifuged at 13,000 rpm at 4 ºC for 10 minutes. The supernatant was kept, and protein was quantified using western immunoblotting. To determine the USP5 protein half-life after SAHA and SE486-11 treatment, Kelly cells were seeded at a density of 1.5 million cells per T75 flask and treated for with DMSO or combination therapy for 24 hours. Twenty-four hours later cells were treated with 100 µg/ml of cycloheximide (Sigma, NSW, Australia) for 0, 3, 6, 12 or 24 hours. Cells were then washed with cold PBS after treatment and harvested. The cell pellet was resuspended in 200 µl RIPA buffer containing protease inhibitor (Sigma-Aldrich, NSW, Australia) and centrifuged at 13,000 rpm at 4ºC for 10 minutes. The supernatant was kept, and protein was quantified using immunoblotting.

**Microscale thermophoresis (MST)**

Instrument and settings: Monolith® NT.115; MST power = 20%; LED power = 40%. USP5 protein, purchased from Sino Biological, was labelled using the NanoTemper Protein Labelling Kit RED-NHS Monolith NT.115 (Amine Reactive) (NanoTemper technologies, Munich, Germany). Labelled protein was diluted to a concentration of 20 nM for all experiments. Compounds were diluted in DMSO, and a serial 1:2 dilution was conducted. The final DMSO concentration in the assay was 5%. Measurements were carried out in MST-buffer (50 mM TrisHCl pH 7.8, 150 mM NaCl, 10 mM MgCl_2_, 0.05% tween 20) and standard capillaries. Data was analyzed using NanoTemper MO Affinity analysis software. Error bars = S.D. from three independent experiments.

**Computational modelling**

All compounds were initially sketched and minimised in MarvinSketch (ChemAxon). The USP5 X-ray crystal structure (PDB:3IHP) was downloaded from the RSCB protein data bank. SAHA was manually docked into this structure using Sybyl X 2.1. (Certara L.P.) to allow docking of the hydroxamate group of SAHA, which was orientated towards the Zn atom of USP5. The TWEAK algorithm was used in Sybyl X 2.1 (Certara L.P.) to search for any alternate loop orientations of amino acids 423-430. These were then manually assessed and compared to the USP7 loop orientation. This modified USP5 structure was then used to dock SE486-11 using standard parameters of the Geom-dock module in Sybyl X 2.1. The top 20 docking solutions were retained, visually analysed and clustered to obtain the proposed binding mode. All figures were created in Pymol (Schrodinger).
